# Investigating the measurement of academic resilience in Aotearoa New Zealand using international large-scale assessment data

**DOI:** 10.1007/s11092-022-09384-0

**Published:** 2022-05-25

**Authors:** Georgia Rudd, Kane Meissel, Frauke Meyer

**Affiliations:** Faculty of Education and Social Work, University of Auckland, Private Bag 92601, Symonds Street, Auckland, 1150 New Zealand

**Keywords:** Academic resilience measurement, Academic resilience operationalisation, Protective factors, Socioeconomic status, International large-scale assessment

## Abstract

Academic resilience captures academic success despite adversity and thus is an important concept for promoting equity within education. However, our understanding of how and why rates of academic resilience differ between contexts is currently limited by variation in the ways that the construct has been operationalised in quantitative research. Similarly, comparing the strength of protective factors that promote academic resilience is hindered by differing approaches to the measurement of academic resilience. This methodological variation has complicated attempts to reconcile disparate findings about academic resilience. The current study applied six commonly used operationalisations of academic resilience that combined different thresholds of high risk and high achievement, to three international large-scale assessments, to explore how these different operationalisations impacted the findings produced. The context of Aotearoa New Zealand was chosen as a case study to further academic resilience research within this context and investigate how academic resilience manifests in an education system with relatively high levels of average achievement alongside low levels of educational equity. Within international large-scale assessment datasets, prevalence rates differed markedly across subject areas, grade levels, and collection cycles, as a function of the measure of academic resilience employed, while the strength of protective factors was more consistent. Thresholds that were norm-referenced produced more consistent findings across the different datasets compared to thresholds that were criterion-referenced. High levels of missing data prevented the analysis of some datasets, and differences in the way that key constructs were measured undermined the comparability of findings across international large-scale assessments. The findings emphasise the strengths and limitations of utilising international large-scale assessment data for the study of academic resilience, particularly within the Aotearoa New Zealand context. Furthermore, the study highlights that researchers’ methodological decisions have important impacts on the conclusions drawn about academic resilience.

**Supplementary Information:**

The online version contains supplementary material available at 10.1007/s11092-022-09384-0.

## Introduction

Academic resilience research explores the concepts of adversity and adaptation within the educational domain to better understand how students at risk of underachievement maintain high levels of academic performance. Students who are academically resilient overcome acute or chronic adversities to demonstrate consistently high levels of achievement, where others do not (e.g. Martin & Marsh, 2008; Wang et al., 1994). Resilience can be contrasted with buoyancy, which focuses on how students overcome the day-to-day challenges associated with education and learning. Despite the definitional simplicity of and general consensus about the concept of academic resilience, there is significant variation in the way that it has been measured (Rudd et al., 2021; Tudor & Spray, 2018; Ye et al., 2021). These variations reflect fundamentally different positions on the information that can be used to infer academic resilience and how the construct should be analysed. For example, academic resilience has been measured both directly, using academic performance measures, and indirectly, by using characteristics indicative of academic resilience (Rudd et al., 2021). There are also different approaches to the analysis of measurement data to investigate academic resilience (Tudor & Spray, 2018). Variable-centred approaches identify protective factors that mediate or moderate the association between indicators of risk and competence, while person-centred approaches employ a categorical variable of resilient and non-resilient sub-samples to explore group differences. Rudd and colleagues (2021) identified three distinct approaches to the measurement of academic resilience (see Table 1) which differed in the way they used data to draw conclusions about academic resilience. The first of these approaches, the definition-driven approach, is the focus of this study.

**Table 1:** Approaches to the measurement of academic resilience

Measurement approach	Description
Definition-driven	Identifies a resilient sub-sample based on predetermined risk and achievement criteria
Process-driven	Investigates interaction of risk and protective factors on an achievement outcome; higher levels of achievement reflect higher levels of resilience
Latent construct	Continuous measure which calculates a resilience score based on characteristics indicative of a student’s capacity for resilience

Adapted from ‘Measuring academic resilience in quantitative research: A systematic review of the literature’ Rudd et al. (2021)

The construction of academic resilience as a categorical, usually binary, variable is common practice (e.g. Borman & Overman, 2004; OECD, 2020b). Rudd and colleagues (2021) labelled this the definition-driven approach, due to its literal application of the academic resilience definition. That is, indicators of high risk and high achievement are identified, with students who meet both of these criteria deemed to be resilient at that point in time. Person-centred approaches to the analysis of academic resilience are then employed to investigate how resilient students differ from their non-resilient counterparts. Within the definition-driven approach, there is great variation in how academic resilience is operationalised. Researchers must choose what indicators of risk and achievement to use and what cut-off scores within these measures will signify academic resilience (Ye et al., 2021). These choices will depend upon the purpose of the study but are ultimately at the discretion of the researcher (Rudd et al., 2021).

Studies that utilise international large-scale assessment (ILSA) data to investigate academic resilience often employ a definition-driven approach (e.g. Cheung, 2017; Sandoval-Hernández & Cortes, 2012), likely because this approach is used by the assessment centres themselves. The Programme for International Student Assessment (PISA) first published findings related to academic resilience in 2011, defining resilient students as those who were both in the bottom third of their national socioeconomic distribution and who achieved in the top third of all PISA participants, accounting for socioeconomic status (SES; OECD, 2011). Key features of ILSAs, including their recurring nature and international representation, facilitate comparisons of academic resilience across contexts: prevalence rates of academic resilience can be compared across countries at a single timepoint or across multiple timepoints for a single country. Accordingly, the data they collect can offer insights about academic resilience that are unlikely to be garnered by any one study alone. For these reasons, the current study focuses on the analysis of ILSA data for the study of academic resilience.

### Measuring the components of academic resilience using ILSA data

There are a variety of circumstances that students may be exposed to that increase their risk of underachievement. Accordingly, academic adversity can be measured in a variety of ways. However, most studies that explore academic resilience, including those using ILSA data, exclusively measure risk using indicators of student background, namely SES (Ye et al., 2021). SES is an important factor in influencing students’ educational experiences. Across Organisation for Economic Co-operation and Development (OECD) countries, 12% of reading performance is explained by students’ SES (OECD, 2020b). Accordingly, SES is one of the most used variables in the PISA datasets, often employed to gain insight into structural inequities within education systems (Avvisati, 2020; Treviño et al., 2021). The importance of SES within educational research has meant that this variable is consistently measured within ILSAs. Thus, its availability as an indicator of academic risk across different collection cycles (i.e. years) makes it useful for operationalising academic resilience across contexts.

Given that ILSAs are underpinned by the collection of achievement data, ILSA data lends itself to a direct measurement approach to academic resilience. In a direct approach, an achievement outcome is used to measure academic success, often in one of the core subject areas (Rudd et al., 2021). ILSAs focus on one or more subject areas: the Progress in International Reading Literacy Study (PIRLS) collects achievement data related to reading, while the Trends in International Mathematics and Science Study (TIMSS) collects both mathematics and science data. Furthermore, PISA assesses three subject areas but identifies a main domain for each collection cycle, which provides a more in-depth understanding about subject specific achievement and its correlates. This means that academic resilience can be measured in relation to a specific subject area, or more broadly, across two or more subject areas. For example, while PISA measures performance in reading, mathematics, and science, the assessment centre operationalises academic resilience exclusively in terms of reading achievement (e.g. OECD, 2020b), whereas other studies require resilient students to achieve highly in multiple domains (e.g. Agasisti et al., 2018). Such differences in resilience criteria reflect different conceptualisations of academic resilience and likely shape the findings produced.

Studies using ILSA data to operationalise academic resilience consistently employ measures of SES and achievement to identify their resilient samples, yet there is still great variation in the way that academic resilience is measured. Ye and colleagues (2021) tested four main operationalisations of academic resilience that applied two different thresholds to the two components of resilience: an absolute or relative threshold of risk and an absolute or relative threshold of achievement. Absolute thresholds remain constant across different contexts, facilitating cross-country comparisons of academic resilience (Rudd et al., 2021; Ye et al., 2021). For example, PISA defines six levels of reading proficiency, based on achievement scores, that are used to describe the complexity of the tasks that a student can complete (OECD, 2019), that is, the levels are criterion-referenced. Agasisti and colleagues (2018) argued that performance at or above proficiency level 3 signified high achievement because it demonstrated that students had developed fundamental skills that would facilitate later success. Similarly, PIRLS and TIMSS define four international benchmarks (low, intermediate, high, and advanced; Martin et al., 2020), which have been used in existing studies of academic resilience (e.g. Erberer et al., 2015). In using absolute thresholds to measure resilience the findings not only reflect the equity of the individual education system of interest, but also the country’s economic and educational standing on the world stage, with wealthier countries more likely to have a higher proportion of their population achieving above a certain proficiency level or benchmark relative to less wealthy countries.

Relative thresholds are oriented towards national studies of academic resilience in which a student’s resilience status will depend on their position relative to their peers. A proportion of the sample is used to determine who is at high risk of underachievement and who is high achieving (i.e. norm-referenced). PISA’s most recent operationalisation of academic resilience identifies resilient students in a nation’s sample as those in the lowest 25% of the socioeconomic distribution, who are also in the top 25% of the achievement distribution (OECD, 2020b). Unlike absolute thresholds, which are sensitive to the international differences in national socioeconomic and achievement distributions, relative thresholds standardise the proportion of students in each education system who will be considered disadvantaged and high achieving. Employing relative thresholds enables the researcher to calculate an ‘optimum’ rate of academic resilience that reflects a truly equitable education system. Thus, using the PISA example, if SES has no bearing on achievement, 25% of students in the lowest SES quartile can be expected to achieve in the highest achievement quartile. Lastly, high achievement can be determined by calculating resilience residuals. The resilience residuals approach, utilised by PISA in earlier collection cycles, is an extension of the relative threshold approach exclusively used to identify high achievement (Rudd et al., 2021). While a proportion of the achievement distribution is still selected, this distribution incorporates the impact that SES has on achievement to identify students who achieve better than expected (i.e. have a positive residual), given their level of risk exposure. Here, high achieving students are those who achieve more highly than other students from similar socioeconomic backgrounds, with prevalence rates below 25% indicating the existence of other risk factors shaping the educational outcomes of students.

Another feature of ILSAs is their collection of contextual information about the student and their educational experiences which can be used to identify factors that increase the likelihood of students demonstrating resilience. These protective factors can be sorted into three developmental contexts: the child, the family, or external (Garmezy, 1991). Factors from these contexts have been studied using ILSA data. For example, Cheung (2017) investigated how four student-level (i.e. child) ‘resilience in learning’ variables from the PISA 2012 cycle predicted academic resilience among five East Asian countries. Students who reported higher levels of self-efficacy, self-concept, and familiarity with mathematical concepts were more likely to be resilient. In addition to student-level characteristics, Agasisti et al. (2018) explored school-level (i.e. external) characteristics associated with resilience in over 50 countries. The strongest school-level predictors were school SES (OR^1^ = 3.7) and disciplinary climate (OR = 2.0). Other studies have explored variables reflective of the family context, such as indicators of parent emotional support, material support, and role modelling by parents, which have been found to promote resilience in Germany, Luxembourg, Belgium, and Sweden (Sandoval-Hernández & Cortes, 2012). Arguably, studying protective factors from multiple developmental contexts offers researchers a more comprehensive understanding of how protective factors individually and collectively facilitate academic resilience. By better understanding what factors increase the likelihood of academic resilience, more students can be supported to develop their resilience by being given the opportunity to engage in a variety of learning experiences and establish pivotal relationships which can be drawn upon when facing adversity.

### The implications of applying different operationalisations of academic resilience

The lack of cohesion among the existing operationalisations of academic resilience prevents the direct comparison of results across studies. Comparisons of rates of resilience across contexts often serve as an indicator of how equitable education systems are. Certainly, within the Aotearoa^2^ New Zealand (NZ) context, rates of academic resilience provide a tangible example of how SES remains an important factor in students’ educational outcomes (e.g. Mutch, 2012; OECD, 2020b). Prevalence rates are typically derived from within the disadvantaged group, rather than as a proportion of the whole sample. This is the most common approach to the reporting of prevalence rates of academic resilience. The use of this approach means that the methodological decisions made about how to measure both high risk and high achievement are important for the interpretation of results. For example, using their national operationalisation of academic resilience (lowest 25% SES; highest 25% achievement), PISA reported that 6.2% of disadvantaged students in Peru demonstrated resilience in reading, while 19.8% of students in Macao (China) did so in the 2018 collection cycle (OECD, 2020b). In contrast, Agasisti et al. (2018) used data from earlier collection cycles and came to very different results. While they used the same definition as PISA for at-risk students, that is those in the lowest 25% of the national socioeconomic distribution, the achievement criterion was defined as reaching PISA proficiency level 3 in reading, mathematics, and science. Using this operationalisation, Peru’s rate of academic resilience was just 0.5%, while 51.7% of disadvantaged students in Macao demonstrated resilience.

Measuring academic resilience in different ways may also impact the strength of the protective factors studied. Indeed, the thresholds used to determine the high risk and high achieving sub-samples will ultimately determine who is resilient and non-resilient. These two groups form the binary outcome used in logistic regression analyses, arguably the most common method of identifying protective factors when academic resilience is operationalised using a definition-driven approach (Rudd et al., 2021). However, even when researchers apply the same types of thresholds to analogous measures of risk and achievement, there are still ways for the measurement of academic resilience to diverge. For example, despite Cheung (2017) and Sandoval-Hernández and Cortes (2012) both using a resilience residuals approach to understand how subject confidence contributed to resilience, their level of threshold for high risk and high achievement differed: Cheung (2017) used the lowest 25% of the SES distribution to identify high risk, compared to the lowest 20% employed by Sandoval-Hernández and Cortes (2012). In addition, Cheung (2017) identified high achievement as scoring in the top 25% of the achievement distribution, compared to the top 20% applied by Sandoval-Hernández and Cortes (2012). Thus, even though both studies investigated the protective strength of subject self-confidence using a resilience residuals approach, the differences in the odds ratios (1.4 and 3.3, respectively), cannot be interpreted without considering the variation in measurement.

### Academic resilience research in Aotearoa New Zealand

Aotearoa NZ is an interesting and important context for academic resilience research, because of its large and persisting educational inequities. While Aotearoa NZ has relatively high levels of average national achievement in PISA tests of reading, mathematics, and science, the country also has one of the largest disparities between their lowest and highest achievers (May et al., 2019). This ‘long tail of underachievement’ is associated with student background (Mutch, 2012), with 12.9% of the variation in achievement being explained by students’ SES (OECD, 2020b). Accordingly, the context of Aotearoa NZ may provide valuable insights into the concept of academic resilience as a case study for how academic resilience manifests in contexts with high levels of academic achievement and low levels of educational equity. In addition, despite consistently participating in ILSAs, few studies have utilised these data for the study of academic resilience within the Aotearoa NZ context. Extant research has typically been conducted by the assessment centres themselves. For example, using their within-country definition, PISA reported that 11.7% of disadvantaged students in Aotearoa NZ demonstrated resilience in the 2018 collection cycle (OECD, 2020b). Thus, the current study will utilise ILSA data to explore variations in the measurement of academic resilience within the Aotearoa NZ context, acknowledging the potential of such findings to provide insights about academic resilience from both a national and international perspective.

### Study aims

The differences in the ways that academic resilience has been operationalised using the definition-driven approach have contributed to disparate findings in the field. However, studies tend to analyse data within ILSAs, across a single (e.g. Cheung et al., 2014) or multiple collection cycles (e.g. Agasisti et al., 2018), rather than *across* ILSAs, raising questions about the comparability of key constructs and findings related to academic resilience. Therefore, more research is required to better identify the sources of variation in the measurement of academic resilience and their contributions to differences in outcomes. This knowledge will help facilitate a more comprehensive understanding of what each measure of academic resilience is capturing and therefore the interpretation and reconciliation of findings across studies. Accordingly, the aim of this study is twofold. Firstly, this study will explore the theoretical and analytical implications of the methodological decisions made by researchers about how to operationalise academic resilience within and across ILSA datasets. Given that studies utilising ILSA data typically employ a definition-driven approach, the current study will apply commonly used absolute, relative, and resilience residuals thresholds to SES and achievement measures and compare findings related to the prevalence rates and protective factors of academic resilience. Thus, the first aim is to probe both the implementation of different operationalisations of academic resilience and the strengths and limitations of utilising various ILSAs for this line of research. Secondly, acknowledging the limited amount of academic resilience research conducted within the context of Aotearoa NZ, this study will provide insight into this phenomenon by focussing on the Aotearoa NZ portions of data within three ILSAs: PISA, PIRLS, and TIMSS. The current study will answer the following research questions:

1. What are the sources of variation in the measurement of academic resilience in quantitative research?
2. What are the impacts of these sources of variation on the findings produced about academic resilience?
3. What are the strengths and limitations of ILSA datasets for the study of academic resilience?
4. What can we learn about academic resilience within the Aotearoa NZ context by analysing recent ILSA datasets?

## Method

### Sample

This study used data from three well-known ILSAs which had conducted at least one data collection cycle since 2015: PISA, PIRLS, and TIMSS. These ILSAs collect achievement data, as well as contextual information about the students and their educational experiences, reported by students, parents, teachers, principals, and administrators. First conducted in 2000 by the OECD, PISA assesses the reading, mathematical, and scientific literacy of 15-year-olds every 3 years (OECD, 2020a). In 2015, the major domain tested by PISA was science, with reading and mathematics the minor domains, thus providing a comprehensive snapshot of science achievement and a trend-level understanding of reading and mathematics achievement. In 2018, the major domain was reading. The PIRLS and TIMSS ILSAs were conducted by the International Association for the Evaluation of Educational Achievement (IEA). TIMSS, first conducted in 1995, is administered every 4 years to students in grades 4 and 8, while PIRLS, inaugurated in 2001, is administered every 5 years to grade 4 students. These ILSAs employ multi-stage sampling procedures, firstly selecting schools and then students within these schools to sit the assessments. Cases are weighted to enable population-level inferences to be made (OECD, 2009). Table 2 outlines the Aotearoa NZ samples employed by the different ILSAs across the collection cycles included in the current study.

**Table 2:** ILSA subjects and Aotearoa NZ samples by collection cycle

ILSA and collection cycle	Subjects	Unweighted sample of Aotearoa NZ students	Weighted sample of Aotearoa NZ students	Technical document
PISA 2015	Reading	4,520	54,274	OECD (n.d.a)
	Mathematics			
	Science			
TIMSS 2015				
Grade 4	Mathematics	6,322	54,217	Martin et al. (2016)
	Science			
Grade 8	Mathematics	8,142	55,931	Martin et al. (2016)
	Science			
PIRLS 2016	Reading	5,646	56,742	Martin et al. (2017)
PISA 2018	Reading Mathematics Science	6,173	53,000	OECD (n.d.b)
TIMSS 2019				
Grade 4	Mathematics Science	5,019	60,098	Martin et al. (2020)
Grade 8	Mathematics Science	6,051	58,038	Martin et al. (2020)

Aotearoa NZ is a small island country in the South Pacific Ocean, southeast of Australia. It has a similar land mass as the UK and a population of approximately five million people (Statistics New Zealand, 2022). In the PISA 2018 collection cycle, Aotearoa NZ’s mean level of achievement were significantly higher than the OECD averages in reading (506 vs. 487), mathematics (494 vs. 489), and science (508 vs. 489). However, the country also had great variation in student outcomes with 165 points separating students scoring in the 5th and 95th percentiles in reading, equating to more than two proficiency levels. A large component of this disparity in achievement was associated with SES. This disparity is despite the country having high levels of average wealth relative to other countries (Shorrocks et al., 2021). Accordingly, Aotearoa NZ is characterised by high levels of average achievement, as well as large disparities between their lowest and highest achievers associated, to a large degree, with student sociodemographic background.

### Measures

#### Indicators of adversity

Each of the ILSAs has created their own composite SES indices which were used to measure students’ level of risk exposure. Using these measures, the current study applied a relative threshold and an absolute threshold to identify the high-risk samples in each of the ILSA datasets, where appropriate.

The PISA Index of Economic, Social and Cultural Status (ESCS) is comprised of three indicators: highest level of parental occupation, highest level of parental education, and number of home possessions (OECD, n.d.a, b; see Appendix A for full list of measures). As the ESCS is a continuous measure, a relative threshold was employed to identify high-risk students. The commonly used lowest 25% threshold was applied which translated to an ESCS cut-off score of − 0.39 for the 2015 collection cycle and − 0.51 for the 2018 collection cycle. PISA does not treat the ESCS as a categorical variable. Accordingly, no absolute threshold was applied to the SES indicator within the PISA datasets.

The Home Resources for Learning scale (HRL) was used by both PIRLS and TIMSS to measure grade 4 students’ SES. It is comprised of five items reflecting parents’ highest level of education and occupation and the availability of four educational resources (Martin et al., 2016, 2017; Yin & Fishbein, 2020). The HRL is a continuous measure of SES which can also be used as a categorical measure, consisting of three groups: few, some, and many resources. Consequently, both relative and absolute thresholds of risk were applied to identify the high-risk samples. The ‘few resources’ category was chosen as the absolute threshold of risk because it represented the lowest socioeconomic bracket and has been used in existing studies (see Table 3 for actual cut-off scores).

**Table 3:** Measures of SES and applied risk thresholds within the Aotearoa NZ context

ILSA and collection cycle	SES measure	Valid cases	Percentage missing	25th percentile	‘Few resources’ cut-off
PISA 2015	ESCS	51,956	4.3%	-0.39	X
TIMSS 2015					
Grade 4	HRL	29,694	45.2%	10.28	7.4
Grade 8	HER	54,688	2.2%	10.19	8.3
PIRLS 2016	HRL	26,726	52.9%	10.22	7.50
PISA 2018	ESCS	51,538	2.8%	− 0.51	X
TIMSS 2019					
Grade 4	HRL	24,696	58.9%	10.26	7.40
Grade 8	HER	56,713	2.3%	9.65	8.40

X = absolute risk not measured

The Home Educational Resources scale (HER) was used to measure the SES of grade 8 students completing the TIMSS assessments (Martin et al., 2016; Yin & Fishbein, 2020). Unlike the ESCS and HRL, the HER does not include a measure of parents’ occupational standing, only capturing levels of parental education and educational resources in the home. Both a relative and absolute threshold was used to identify the high-risk samples.

Despite the assessment centres imputing missing SES data where possible (Martin et al., 2017), over half of the PIRLS 2016 and TIMSS 2019 grade 4 datasets did not contain valid SES data, while the TIMSS 2015 grade 4 dataset was missing over 45% of SES data. Given that SES is a key component required for the measurement of academic resilience, this is a significant amount of missing data. The incomplete representation of the SES distribution means that it is likely that using these datasets would not accurately capture the students who are most at risk of underachievement based on their socioeconomic circumstances. As a result, the prevalence rates and strength of protective factors were not analysed for the PIRLS 2016 and TIMSS 2015 and 2019 grade 4 datasets. All subsequent measures and analyses relate to the PISA 2015 and 2018 and TIMSS 2015 and 2019 grade 8 datasets only.

#### Achievement measures

Academic resilience was investigated in the areas of reading (PISA), mathematics (PISA and TIMSS), and science (PISA and TIMSS). Plausible values were used to measure achievement at the population-level. Plausible values are created by calculating posterior distributions of possible achievement scores for each participant which are imputed based on their assessment responses and background characteristics using item response theory (OECD, 2009). Plausible values are then drawn randomly from the posterior distributions. All plausible values were used to calculate the absolute and relative achievement thresholds, while resilience residuals were calculated using the first subject plausible value only.

Relative, absolute, and resilience residuals thresholds were calculated to identify high achievement (see Table 4). Firstly, the top 25% threshold of the national achievement distribution for each ILSA dataset was calculated to provide the relative achievement thresholds. Both PISA and TIMSS have their own international benchmarking systems which were employed as the absolute thresholds of achievement in the current study. The chosen thresholds of PISA proficiency level 3 and TIMSS intermediate benchmark have been used in existing studies (e.g. Agasisti et al., 2018; Erberer et al., 2015). Resilience residuals were also calculated to identify high achievement. Resilience residuals were derived by regressing subject achievement on the relevant SES measure. Standardised residuals were then calculated for each student, and the 75th percentile of these residuals was identified to provide the high achievement threshold.

**Table 4:** Achievement cut-off scores for the relative, absolute, and resilience residuals achievement thresholds

	Reading	Mathematics	Science
Relative achievement thresholds			
PISA 2015	584.18	559.97	587.71
PISA 2018	584.27	560.09	582.41
TIMSS 2015	X	555.49	576.39
TIMSS 2019	X	541.62	562.83
Absolute achievement thresholds			
PISA 2015	480	482	484
PISA 2018	480	482	484
TIMSS 2015	X	475	475
TIMSS 2019	X	475	475
Resilience residuals achievement thresholds			
PISA 2015	0.68	0.69	0.66
PISA 2018	0.73	0.68	0.71
TIMSS 2015	X	0.68	0.67
TIMSS 2019	X	0.67	0.66

X = achievement not tested

**Fig. 1:**
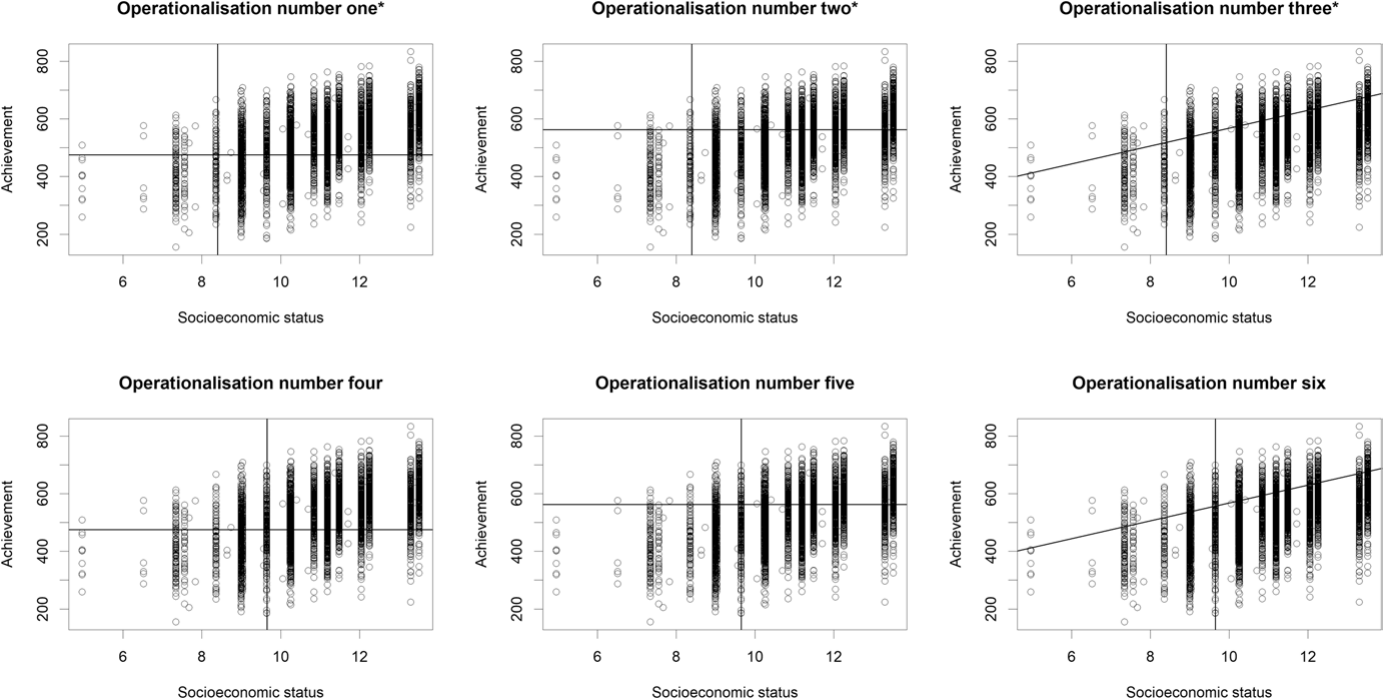
Graphical representation of the six operationalisations used in the current study. * = not applied to PISA datasets. TIMSS 2019 grade 8 dataset used as an example. The first science achievement plausible value was used as the proxy for achievement

Combining the two measures of high risk and three measures of high achievement resulted in six operationalisations of academic resilience (see Table 5). Figure 1 illustrates these operationalisations graphically. The high-risk sample is identified as those to the left of each graph, with students obtaining high achievement scores shown in the top section of each graph. Thus, academic resilience is captured in the top left-hand quadrant of each graph.

**Table 5:** The six operationalisations of academic resilience used in the current study

Risk thresholds	Achievement thresholds		
	Absolute	Relative	Resilience residuals
Absolute	1. Few resources on SES measure; intermediate benchmark of achievement *	2. Few resources on SES measure; highest 25% achievement *	3. Few resources on SES measure; highest 25% achievement, accounting for SES *
Relative	4. Lowest 25% SES; proficiency level 3 (PISA) or intermediate benchmark of achievement (TIMSS)	5. Lowest 25% SES; highest 25% achievement	6. Lowest 25% SES; highest 25% achievement, accounting for SES

* = not applied to PISA datasets

#### Protective factors

It was intended that the protective factors in the current study would capture similar constructs across the PISA and TIMSS ILSAs to facilitate cross-dataset comparisons. However, very few questionnaire items and derived variables were comparable. Consequently, two sets of protective factors were chosen to facilitate comparisons within and across the different ILSA collection cycles. Table 6 outlines each set of protective factors, which include three measures collected and analysed at the student-level, reported by the student, and three measures collected and analysed at the school-level, reported by the school principal or administrator, which attempt to capture protective factors from all three developmental contexts. The aim of facilitating within-dataset comparisons was balanced with the use of subject-specific protective factors that were hypothesised to be most relevant to resilience in the different subject areas. For PISA, this was dictated by the main domain of the particular collection cycle of interest, whereas for TIMSS subject-specific measures were captured separately for mathematics and science and used where appropriate (i.e. mathematics confidence was used to predict resilience in mathematics). For derived protective factors, the assessment centres required students to have at least two (TIMSS; Martin et al., 2017) or three (PISA; OECD, n.d.a, b) valid responses to the scale’s indicator items to have a score imputed for that measure. Cases that did not meet this threshold were not included in the logistic regression analyses.

**Table 6:** Definition of the protective factors and control variables used in the current study

	PISA		TIMSS	
	Protective factor	Definition	Protective factor	Definition
Student-level	Subject confidence	Students’ assessment of how well they can achieve subject-specific tasks	Subject confidence	Students’ assessment of how well they can achieve in a specific subject area
	Parental support	Students’ perceived level of emotional support from parents	Homework	The amount of time students spend on completing assigned homework
	School belonging	Student’s sense of school belonging	School belonging	Student’s sense of school belonging
School-level	Extracurricular activities	The number of creative extra-curricular activities offered by the school	Curriculum implementation	The degree to which principals believe their teachers are successful in implementing the school’s curriculum
	Student–teacher ratio	The number of students a teacher is responsible for when the student roll is equally distributed across the total number of teachers within a school	Teacher expectations	The degree to which principals believe their teachers hold high expectations for student achievement
	Professional development	The percentage of the teaching staff within a school who had attended a programme of professional development over the last three months	Parent expectations	The degree to which principals believe their students’ parents hold high expectations for student achievement
Control variables	Gender	Students’ self-reported gender	Gender	Students’ self-reported gender
	School SES	The estimated proportion of students within Year 10 that come from ‘socioeconomically disadvantaged homes’	School SES	The estimated proportion of students within the school that come from ‘socioeconomically disadvantaged homes’

#### Control variables

The current study sought to identify protective factors that were amenable to change, in order to provide actionable information that could be used to facilitate resilience. Acknowledging that achievement is influenced by both gender and school SES, circumstances that are typically stable over time, these variables were treated as covariates in the logistic regression models (see Table 6).

### Data analysis

All analyses were conducted using the IEA International Database Analyzer (IDB Analyzer; IEA, 2019). Developed specifically to analyse ILSA data, the IDB Analyzer generates IBM SPSS Statistics syntax that accounts for the specific survey design, such as the use of sampling weights, replicate weights, and plausible values, to produce accurate parameter estimates and standard errors. Total student sampling weights were applied to enable population-level inferences to be made, while replicate sampling weights were used to produce standard errors that accounted for the complex designs of the ILSAs, such as sample clustering (Birrell et al., 2019; Hahs-Vaughn, 2005; Jerrim et al., 2017; Thomas & Heck, 2001).

The IDB Analyzer was used in three stages of data preparation and analysis. Firstly, the relative risk thresholds and the relative and resilience residuals achievement thresholds were identified by calculating the 25th and 75th percentiles, respectively. Secondly, the proportion of students within the at-risk samples who met the nominated achievement thresholds was calculated to provide the prevalence rates of academic resilience produced by each operationalisation. Lastly, logistic regression models were fitted to estimate the likelihood of being resilient using the outlined protective factors. The sample was narrowed to those students who met the relevant high-risk threshold. The protective factors and control variables were then entered into the model, with the binary achievement outcome created by applying the nominated high achievement threshold. The binary outcome was comprised of those identified as resilient (1) and all other disadvantaged students not identified as resilient (0).

## Results

Six operationalisations were explored by applying commonly used absolute, relative, and resilience residuals thresholds to identify high risk and high achievement sub-samples. Students who met both of these criteria were deemed to be resilient. Firstly, we present the findings in relation to the prevalence rates of academic resilience produced by the different operationalisations within two ILSAs and across two collection cycles. Given that all operationalisations include a measure of adversity, the reported prevalence rates can be interpreted as the proportion of disadvantage students who demonstrate high achievement, as defined by the nominated achievement threshold. Secondly, the odds ratios of the logistic regression analyses are presented. Here, odds ratios are used to compare the strength of the protective factors studied to identify what factors significantly increase the likelihood of a disadvantaged student demonstrating resilience.

### Prevalence rates of academic resilience

The impact of how academic resilience is operationalised is demonstrated in the wide variation in prevalence rates presented in Table 7. Overall, rates of resilience ranged from 2.6 to 43.3% of students identified as being at a heightened risk of underachievement. The first three operationalisations were applied only to the TIMSS datasets which included an absolute threshold of risk. Thus, the differences in the prevalence rates for a specific subject area (e.g. science) within a collection cycle (e.g. 2015) demonstrate the impact of applying the different high achievement thresholds. The absolute achievement benchmark was much lower than the relative achievement benchmark, resulting in a higher rate of resilience (28.3% vs. 2.6%). The resilience residuals approach also increased the rate of resilience (29.5%) by adjusting the achievement benchmark based on students’ exposure to low-SES circumstances. Interestingly, in the first two operationalisations, the subject with the higher rate of academic resilience differed: when an absolute measure of achievement was used, higher resilience rates were found for students in science, whereas when a relative measure was used, higher resilience rates were found for students in mathematics. Across the first three operationalisations of academic resilience explored here, the rates of resilience generally increased over time, with the exception of the TIMSS science datasets analysed using operationalisation number one. The resilience residuals approach garnered similar results within and across subject areas and collection cycles, perhaps indicating the steadiness of the association between SES and achievement.

**Table 7:** Prevalence rates of academic resilience

	1. Absolute risk; absolute achievement	2. Absolute risk; relative achievement	3. Absolute risk; resilience residuals	4. Relative risk; absolute achievement	5. Relative risk; relative achievement	6. Relative risk; resilience residuals
PISA 2015						
Reading	X	X	X	43.3%	10.9%	23.7%
Mathematics	X	X	X	34.9%	10.3%	23.0%
Science	X	X	X	40.7%	10.0%	23.3%
TIMSS 2015						
Mathematics	20.7%	4.0%	28.9%	29.1%	6.5%	21.7%
Science	28.3%	2.6%	29.5%	37.4%	5.2%	22.5%
PISA 2018						
Reading	X	X	X	42.2%	11.7%	25.3%
Mathematics	X	X	X	36.3%	11.0%	24.7%
Science	X	X	X	40.9%	11.2%	25.5%
TIMSS 2019						
Mathematics	23.5%	6.1%	29.3%	29.1%	8.7%	23.8%
Science	26.2%	4.6%	28.4%	35.4%	7.0%	25.7%

X = absolute risk not measured. Percentages reflect the proportion of the identified at-risk sample that demonstrate high achievement (i.e. academic resilience)

The final three operationalisations of academic resilience were applied to both collection cycles of the PISA and TIMSS ILSAs. These operationalisations used a relative threshold of risk to identify the disadvantaged group. That is, students needed to be in the lowest 25% of the national SES distribution to be considered at a heightened risk of underachievement. Operationalisation number four applied an absolute threshold of achievement which differed across the two ILSAs. Therefore, differences between ILSAs were to be expected. Indeed, the TIMSS datasets nearly always produced lower rates of resilience when comparing like subject areas compared to the PISA datasets, suggesting that the TIMSS intermediate achievement benchmark reflects a higher level of subject achievement than the PISA proficiency level 3 benchmark. However, within ILSAs, prevalence rates were fairly consistent both within subject areas and across the two collection cycles. This indicates that a similar proportion of disadvantaged students achieved at levels deemed to be sufficient for facilitating future success, across the two cohorts and different subject areas investigated.

Compared to the first four operationalisations of academic resilience, which included at least one absolute threshold of risk and/or achievement, the final two operationalisations exclusively used relative and/or resilience residuals thresholds to measure high risk and high achievement (i.e. norm-referenced thresholds). Arguably, these operationalisations produced the most stable prevalence rates of academic resilience within ILSAs, regardless of subject area. For example, the difference in prevalence rates for reading, mathematics, and science achievement within the PISA 2018 collection cycle was just 0.7%. The TIMSS datasets produced similarly comparable rates of resilience, albeit these were somewhat smaller than those produced by the PISA datasets. For example, while 10% of students in the lowest 25% of the SES distribution achieved in the top 25% of the science achievement distribution in the PISA 2015 collection cycle, just 5.2% of students who participated in the TIMSS 2015 data collection cycle did so. Similar to the first three operationalisations explored using the TIMSS datasets (that included an absolute risk threshold), rates of resilience derived from operationalisation number five increased very slightly from one collection cycle to the next.

Finally, operationalisation number six (lowest 25% SES; highest 25% achievement, accounting for SES) produced prevalence rates that behaved in a similar manner to those produced by operationalisation number five. Given that this operationalisation accounts for the impact of SES within the measure of academic resilience itself, it is not surprising that these rates were much higher than those produced by operationalisation number five. However, just three out of the ten prevalence scores reached the 25% threshold within this measure. This suggests that while SES does have a strong association with achievement, there are other factors not modelled here which have an influence on students’ achievement. Overall, within datasets and collection cycles, rates of resilience were fairly consistent. This was especially true among measures that exclusively used relative and/or resilience residuals thresholds. However, there was great variation between the six operationalisations employed, demonstrating the impact that the methodological choices of researchers have on the subsequent results produced.

### Protective factors predicting academic resilience

Logistic regression analyses were firstly conducted on the PISA datasets to explore the strength of protective factors, employing operationalisations four, five, and six. These operationalisations used a relative measure of low-SES to identify the sample of disadvantaged students. Regardless of the operationalisation employed, subject confidence (which differed between collection cycles depending on the main domain of interest) was always significant and positively predicted resilience. Students who had high levels of science self-efficacy or reading competence were nearly 3 times more likely to be resilient than students who had low levels of subject confidence. The control variable of school SES was also a consistent predictor of resilience, whereas the other control variable, gender, was only sometimes significant and the direction of this association varied depending on the subject area. Very rarely were any other protective factors found to be statistically significant: in one case, school belonging negatively predicted resilience (see Table 8), while in another, a higher student–teacher ratio (i.e. larger class sizes) positively predicted resilience for students in mathematics (see Table B3). However, it should be noted that the odds ratio for this variable was small (1.09), indicating while the result was significant, the relationship was marginal. Interestingly, the difference in measure of subject confidence across the collection cycles was not obvious in the findings produced. That is, the odds ratios of science self-efficacy in predicting reading and science achievement were comparable (1.34 and 1.37 when applying an absolute achievement threshold). A similar pattern was found for reading competence (2.92 and 2.36, respectively), although the odds ratios for subject confidence were much higher for all subject areas in the 2018 dataset compared to 2015. Full model statistics for all logistic regression models can be found in Appendix B.

**Table 8:** Odds ratio for protective factors predicting academic resilience for the PISA science datasets

	4. Lowest 25% SES; proficiency level 3		5. Lowest 25% SES; highest 25% achievement		6. Lowest 25% SES; highest 25% achievement, accounting for SES	
	PISA 2015	PISA 2018	PISA 2015	PISA 2018	PISA 2015	PISA 2018
Control variables						
Gender	0.95	0.71*	0.86	0.83	0.86	0.73*
School SES	0.98**	0.98***	0.98**	0.98***	0.98**	0.98***
Student-level protective factors						
Subject confidence	1.37***	2.36***	1.65***	2.24***	1.45***	2.06***
Parental support	1.07	1.03	1.02	1.01	0.91	1.11
School belonging	0.91	0.89	0.89	0.82	0.79	0.82*
School-level protective factors						
Extracurricular activities	1.03	1.10	1.07	1.25	0.89	1.06
Student–teacher ratio	0.99	0.99	1.06	0.96	1.06	0.98
Professional development	1.00	1.00	1.01	1.00	1.00	1.00

*Note.* See Appendix B for full model statistics

^*^p < .05. **p < .01. ***p < .001

When academic resilience was operationalised using the lowest 25% of the TIMSS SES distribution, a similar pattern emerged. In all three of the operationalisations (four, five, and six) employed across the two subjects and two collection cycles, subject confidence consistently predicted resilience. Disadvantaged students were up to 1.90 times more likely to be resilient if they had high levels of subject confidence compared to students with low levels of subject confidence. Students’ sense of school belonging was predictive only for operationalisation number six, when applied to the TIMSS 2019 dataset in science (OR = 1.10; see Table 9). The final student-level variable, time spent on assigned homework, was also statistically significant, although only in the 2015 datasets. Spending more time on homework increased the likelihood of resilience by up to 1.41 times across four of the operationalisations and subject areas tested. Similar to the PISA datasets, school SES was always significant, while gender was only significant on one occasion.

**Table 9:** Odds-ratio for protective factors predicting academic resilience for the TIMSS science datasets

	4. Lowest 25% SES; intermediate benchmark		5. Lowest 25% SES; highest 25% achievement		6. Lowest 25% SES; highest 25% achievement, accounting for SES	
	TIMSS 2015	TIMSS 2019	TIMSS 2015	TIMSS 2019	TIMSS 2015	TIMSS 2019
Control variables						
Gender	0.93	1.04	1.16	0.72	0.96	0.64**
School SES	0.63***	0.65**	0.67*	0.60**	0.75**	0.71**
Student-level protective factors						
Subject confidence	1.27***	1.25**	1.42***	1.42***	1.24***	1.32***
Homework	1.24**	1.02	1.31	1.06	1.21**	1.04
School belonging	1.00	1.04	1.01	1.06	0.98	1.10**
School-level protective factors						
Curriculum implementation	0.96	0.98	0.80	0.63	0.87	0.90
Teacher expectations	0.69	0.85	0.65	0.99	0.72	1.09
Parent expectations	0.84	1.11	0.99	1.00	0.94	0.90

*Note.* See Appendix B for full model statistics

^*^p < .05. **p < .01. ***p < .001

Finally, logistic regression models were applied to the TIMSS datasets, this time utilising the first three operationalisations of academic resilience, that included an absolute measure of high risk, as the outcome variable. Results were varied across the achievement outcomes and operationalisations tested. While five out of the 12 regression models identified one significant predictor of resilience (subject confidence), in four of the regression models, none of the protective factors or control variables were significant (see Table 10). This may be reflective of the smaller sample sizes associated with using the absolute measures of high risk explored in the current study. While subject confidence was mostly significant, the strength of this protective factor ranged from 1.40 to 2.33, seemingly a larger variation than the other datasets and operationalisations tested in the current study. Furthermore, time spent on assigned homework was predictive in two instances. In particular, students who spent more time on science homework were 3.30 times more likely to be resilient than students who did not dedicate time to completing assigned homework when operationalisation number two was applied to the TIMSS 2015 science dataset. This was a much higher odds-ratio than those associated with subject confidence and was the only model within the TIMSS 2015 datasets in which subject confidence did not predict resilience. Overall, the strength of protective factors remained remarkably consistent across the six operationalisations tested, with a greater stability in findings produced by the three operationalisations that utilised a relative measure of high risk, compared to the three operationalisations that utilised an absolute measure of high risk.

**Table 10:** Odds-ratio for protective factors predicting academic resilience for the TIMSS science datasets

	1. Few resources SES; intermediate benchmark		2. Few resources SES; highest 25% achievement		3. Few resources SES; highest 25% achievement, accounting for SES	
	TIMSS 2015	TIMSS 2019	TIMSS 2015	TIMSS 2019	TIMSS 2015	TIMSS 2019
Control variables						
Gender	0.80	1.06	1.46	1.22	0.81	0.76
School SES	0.79	0.71	0.87	0.68	0.99	0.81
Student-level protective factors						
Subject confidence	1.46*	1.30	1.37	1.43	1.40*	1.12
Homework	1.38	0.93	3.30***	0.52	1.54*	0.98
School belonging	0.97	0.89	1.05	0.87	0.94	0.93
School-level protective factors						
Curriculum implementation	1.13	1.08	0.65	0.54	1.05	1.11
Teacher expectations	0.70	0.81	0.81	1.51	0.65	0.81
Parent expectations	0.86	0.61	1.58	0.39	0.94	0.77

*Note.* See Appendix B for full model statistics

^*^p < .05. **p < .01. ***p < .001

## Discussion

The operationalisation of academic resilience involves several decisions about how to measure risk and achievement (Ye et al., 2021). These decisions ultimately determine who is and is not identified as resilient and therefore who academic resilience findings are based upon. The current study applied six operationalisations of academic resilience commonly employed by studies using ILSA data to explore how the way in which academic resilience is measured is reflected in the resulting prevalence rates and strength of protective factors studied. Given that there is currently no consensus as to the gold standard of academic resilience measurement (Tudor & Spray, 2018), investigating the theoretical and analytical implications of these operationalisations was intended to broaden the knowledge base about the methodological decisions and outcomes associated with academic resilience to ensure that the concept is accurately represented in quantitative research. The current study also attempted to align the methodological decisions related to the study of academic resilience across different ILSAs to reconcile the findings produced and better understand the strengths and limitations and potential contributions of studies, which analyse ILSA data, to the resilience field. Finally, the current study sought to gain insight into the phenomenon of academic resilience within the Aotearoa NZ context.

### Sources of variations in the measurement of academic resilience

Within the definition-driven approach to the measurement of academic resilience, there are multiple sources of variation that a researcher must consider when deciding how to operationalise the construct. The current study concentrated on two of these sources of variation within and across ILSAs: how to measure risk and achievement and the type of threshold used to discern high risk and high achievement. A third source of variation, the level of threshold applied, was not tested. The first source of variation is the actual measures of risk and achievement. Within the three ILSAs used in the current study, all had created their own SES index. There was a high level of agreement about what types of indicators should be used to measure SES (parental education, parental occupation, and an index of home resources as a proxy for income). This reflected the traditionally ‘tripartite nature of SES’ (Sirin, 2005, p. 418). However, how these indicators were measured differed. In particular, there was great variation in the number of items used to capture the educational resources in the home. This raises the question of whether all three ILSAs were tapping into each of the three components of SES to the same degree and thus whether the findings produced using these measures can be directly compared (i.e. construct validity). Indeed, various researchers have criticised PISA’s ESCS measure and called for revisions to be made to address validity and reliability issues (Avvisati, 2020), such as the inaccuracy of student-report data related to their parents’ level of education (Schultz, 2006).

ILSAs must also address the challenges associated with creating a single SES measure that is relevant to multiple and culturally varied contexts (Rutkowski & Rutkowski, 2013). Treviño et al. (2021) tested the metric and scalar invariance of SES measures from three ILSAs, including PISA, and found that none of these datasets reached scalar invariance and just one study, the Third Regional Comparative and Explanatory Study, achieved metric invariance. Thus, the ESCS, for example, may be more effective in capturing SES in some contexts compared to others, including Aotearoa NZ, due to differences related to how the three components of parental education, parental occupation, and home resources are measured by researchers and interpreted by respondents (e.g. differences in the prestige of certain job titles across contexts). Consequently, despite different ILSAs purporting to measure similar constructs, such as SES, individual differences in the way these constructs are measured and their relevance to the specific context of interest will likely manifest in the findings produced. This arguably prevents the direct comparison of results across ILSAs, and potentially within ILSAs (i.e. across education systems) as well.

The second source of variation is the type of threshold applied. Three different thresholds were investigated: absolute, relative, and resilience residuals. These thresholds reflect different conceptualisations of resilience, which likely lend themselves to specific study contexts. The absolute thresholds of risk and achievement employed by the current study were created by the assessment centres themselves. The ‘few resources’ category of the HER scale (used to measure SES in the TIMSS grade 8 datasets) captured those students who scored the lowest possible score on each of the three SES indicators. This threshold provides a clear description of the types of challenges these ‘at-risk’ students are facing. Similarly, the absolute achievement thresholds were determined by criteria that specify the level of subject skills and knowledge students in these bands are typically capable of (see technical documents outlined in Table 2). These criterion-referenced thresholds put students from around the world on an equal footing, allowing comparisons to be made across different contexts, and give context to the measures of resilience employed. Applying such thresholds to operationalise academic resilience implicitly signals a wider expectation of what we want all students, including those at a disadvantage, to be able to achieve.

In contrast, the use of relative thresholds means that students’ resilience statuses are dependent on their position within the national risk and achievement distributions. The cut-off scores do not have meaning in and of themselves. Thus, the experiences of adversity and levels of achievement demonstrated by resilient students are not easily interpreted in absolute terms (Ye et al., 2021). Using relative thresholds also means that students experiencing similar levels of socioeconomic disadvantage may not be categorised in the same way. However, relative thresholds acknowledge that high risk and high achievement will look different depending on the context. By defining these criteria in terms of relativity to one’s peers means that students that are considered both high risk and high achieving are represented in all contexts (OECD, 2011). This is an important sentiment of academic resilience research because it emphasises that there are students demonstrating academic resilience within every education system. It is this strength-based lens that distinguishes this line of work from studies that address educational inequities associated with SES using a deficit framework, such as by framing the individual as the ‘problem’ or suggesting solutions based on successful models of development derived from a narrow section of society (typically middle-class European samples; Davis-Kean, 2005; Morales & Trotman, 2011). Thus, relative thresholds, which standardise the proportion of high risk and high achieving sub-samples required to identify resilient students, facilitate a within-country exploration of academic resilience, including the study of culturally relevant protective factors (OECD, 2011).

Lastly, the resilience residuals approach, exclusively used to identify high achievement, acknowledges that exposure to a greater amount of risk will likely result in more barriers to achievement. This approach incorporates the impact of the identified risk factor when measuring achievement in order to capture individuals who achieve comparatively well. Thus, especially in more inequitable education systems where there is a strong association between SES and achievement, applying a resilience residuals threshold will result in a larger and potentially more consistent proportion of students demonstrating resilience than approaches that do not account for risk concurrently.

### The impact of the variations in measurement on the findings produced about academic resilience

The multiple operationalisations derived from the variations in the conceptualisation and measurement of high risk and high achievement contribute to differences in outcomes related to academic resilience. In the current study, the prevalence rates ranged from 2.6 to 43.3% across the six operationalisations investigated. This was a smaller range than that found by Ye and colleagues (2021) in Hong Kong, Norway, and Peru, potentially reflecting the currently low levels of educational equity related to SES within the Aotearoa NZ context. The most consistent rates of resilience were produced when the norm-referenced thresholds of risk and achievement were combined (operationalisation number five and six). It may be that assessing one’s resilience status in relation to others may nullify some of the differences in the ways that risk and achievement are measured over time and provide more reliable estimates of academic resilience within ILSAs. That is, using relative thresholds may increase the comparability of resilience findings by reducing the need to use the same exact measures because, even when SES is operationalised in different ways, for example, a relative threshold will likely capture similar groups of people (i.e. at the lower end of the SES distribution). A relative risk threshold also standardises the size of the at-risk population, allowing changes in the rates of academic resilience to be compared over time, without needing to account for changes in the overall wealth of the country. Due to the sources of variation in the measurement of academic resilience previously acknowledged, variations in the rates of academic resilience were expected across ILSAs. However, even within ILSAs, where measures of SES and achievement are purportedly analogous, there were significant differences in the rates of resilience across operationalisations, emphasising the ways in which the methodological decisions of researchers can shape the subsequent outcomes produced.

Whereas the proportion of disadvantaged students demonstrating resilience varied by operationalisation, the strength of protective factors did not. The findings were almost entirely consistent within ILSAs, both across operationalisations (namely, operationalisations four, five, and six) and protective factors. In particular, across the regression models that measured academic resilience using a relative threshold of risk (i.e. the lowest 25% of the SES distribution), subject confidence was always a statistically significant predictor of academic resilience, even when controlling for the other student- and school-level protective factors and control variables. School SES also consistently predicted resilience, while gender did so in about a third of the regression models, although the direction of this association depended on the subject area. Rarely were any of the other protective factors associated with resilience and when this did occur, there was no obvious pattern to these findings. When academic resilience was operationalised using an absolute measure of risk (i.e. ‘few resources’ on the HER scale), results were less consistent. Where subject confidence did predict resilience the odds ratios were more variable within subject areas compared to the regression models that measured academic resilience using a relative threshold of risk. Ye and colleagues (2021) suggest that the stability in the strength of protective factors across operationalisations may reflect some degree of consistency in the way in which academic resilience is measured and is, thus, identifying similar groups of resilient students on which the conclusions about the phenomenon are based upon.

### The strengths and limitations of ILSA datasets for the study of academic resilience

The current study employed publicly available ILSA data for the study of academic resilience. The accessibility, convenience, and reduced data collection costs for individual researchers of these vast datasets make them appealing for educational research (Vartanian, 2011), including resilience research. Indeed, the three ILSAs of interest in the current study had reportedly collected two key components required to measure academic resilience (risk and achievement), as well as a multitude of potential protective factors in the form of contextual variables. This combination of variables, alongside the recruitment of a nationally representative sample, is rare within the Aotearoa NZ context and thus enables an exploration of resilience unlikely achievable by any other means. Furthermore, the repeated nature of collection cycles facilitates the comparison of academic resilience findings over time. In particular, comparing the prevalence rates of academic resilience, derived from operationalisation number five (lowest 25% SES; highest 25% achievement), provides a way in which to determine whether achievement outcomes related to SES are becoming more equitable within an education system. That is, comparing prevalence rates across collection cycles can be used to increase levels of accountability among policy makers, school administrators, principals, and teachers to ensure that students are receiving a high-quality education and experiencing educational opportunity and success irrespective of their socioeconomic background. Thus, the breadth of data within collection cycles and their reoccurring nature of ILSAs over time provide a unique opportunity to study academic resilience both within and across contexts.

However, the limitations of conducting secondary data analysis are obvious; in particular, that an independent researcher has no control over what and how much data has been collected (Smith, 2008). Indeed, as discussed earlier, the first source of variation in the measurement of academic resilience is the actual measures of SES and achievement used to operationalise risk and achievement. The variation in the measurement of these components across ILSAs raises questions as to whether it is legitimate to compare the prevalence rates of and strength of protective factors related to academic resilience across datasets. Another constraint of using ILSA data was the large amount of missing data. SES data was available for less than 60% of the PIRLS 2016 and TIMSS 2015 and 2019 grade 4 datasets which compromised the representation of the national SES distributions and significantly reduced the sample of low-SES students from which the conclusions about academic resilience would be based upon. This ultimately resulted in these three datasets being excluded from analysis altogether. Considering that missing data was only a problem for Aotearoa NZ and three other countries who participated in the PIRLS 2016 assessments (Mullis et al., 2017), this may only be a key reason preventing the analysis of existing ILSA data for the study of resilience within the Aotearoa NZ context. Thus, future collection cycles must find ways to ensure that adequate amounts of contextual data are collected alongside the compulsory tests of achievement to ensure that the full potential of ILSAs can be realised.

The study of protective factors is an important component of academic resilience research, providing actionable information to help address educational inequities. While the breadth of contextual data facilitates the identification of factors that promote resilience, the nature of ILSAs also somewhat limits this exploration. Firstly, both the PISA and TIMSS datasets did not collect parent-level data. For the PISA datasets, a parent questionnaire was created but not completed by the Aotearoa NZ sample, while TIMSS did not create a parent questionnaire for its grade 8 student cohort. The implication of this lack of data is the same: findings about parent behaviour and attitudes and the home environment that may facilitate resilience can only be gleaned from external sources, such as students, which may reflect individual differences among the people completing the questionnaire, as much as what they are reporting on. For example, in the PISA datasets, parental support is reported by the student, thus capturing their interpretation of their parents’ provision of emotional support. Secondly, ILSAs are typically cross-sectional in nature which prevents the exploration of causal pathways related to academic resilience (Ye et al., 2021). Longitudinal datasets can facilitate such investigations, as well as documenting how one’s levels of academic resilience may change as they age or are exposed to new and varied academic adversities and protective factors. Thus, longitudinal datasets may offer a more in-depth understanding of academic resilience by tracking students’ resilience statuses over time and the changing impact of protective factors across the lifespan.

### Resilience in the Aotearoa NZ context

The current findings reflect the large disparities in achievement within the Aotearoa NZ education system and the contribution that SES makes to these disparities. Most notably, when both the relative thresholds of risk and achievement were used (operationalisation number five), Aotearoa NZ’s rates of resilience were well below the desired 25% rate, which would indicate equal distribution of achievement across the SES quartiles. Given the potential for the COVID-19 pandemic to exacerbate existing inequities within the education system (Te Ihuwaka: Education Evaluation Centre, 2021), improving Aotearoa NZ’s rates of academic resilience is becoming an increasingly relevant approach to addressing the country’s ‘long tail of underachievement’. The results of applying operationalisation number five, the same operationalisation as that currently used by PISA, across two ILSAs and three subject areas also suggested that in Aotearoa NZ, rates of academic resilience only increased very slightly between collection cycles (i.e. since 2015). This emphasises the consistent and pervasive impact that SES has on achievement. However, when SES was accounted for in operationalisation number six, there was still some variation in the prevalence rates of academic resilience, and not all prevalence rates met the expected 25% rate. Thus, it is important to note that SES is not the only structural risk factor that is associated with achievement in the Aotearoa NZ context and suggests that other risk factors, such as migration background and between-school variances in quality and achievement, should be explored to address educational disparities (UNICEF, 2018).

Findings from the logistic regression models indicate that the student-level variable, subject confidence, was the most important protective factor for increasing the likelihood of resilience for students in Aotearoa NZ. This aligns with international research suggesting that confidence and other non-cognitive variables, such as self-concept and subject enjoyment, are positively associated with achievement and resilience (e.g. Cheung et al., 2014; Cheung, 2017; Nicholson et al., 2013; Stankov et al., 2012, 2014). One interpretation of this protective factor is that rather than facilitating resilience, subject confidence is the result of high achievement. It is more likely that confidence and achievement have reciprocal effects (e.g. Ganley & Lubienski, 2016; Talsma et al., 2018). Thus, while there is still debate as to whether confidence causes or is caused by high achievement, it is clear that there are differences in the levels of subject confidence between resilient and non-resilient students in Aotearoa NZ and efforts should be made to ensure that students are given opportunities to develop their confidence in different subject areas. Finally, while school SES was treated as a control variable, its consistency as a predictor of resilience suggests that the proportion of low-SES students within a school is negatively associated with disadvantaged students’ likelihood of achieving highly. Policy makers must ensure that schools are being resourced effectively to meet the needs of their community and deliver equitable educational outcomes for students.

### Limitations and future directions

The current study sought to investigate operationalisations of academic resilience typically used in the analysis of ILSA data by employing measures of SES as the indicator of academic risk. However, risk related to achievement is much broader than standard measures of SES encapsulate. Different types of risk may impact students’ educational experiences, attitudes, and behaviours in different ways. Thus, students from different risk backgrounds may navigate different pathways to resilience. Ye and colleagues (2021) found that rates of resilience differed depending on which measure of human capital was used to measure SES, while other studies of academic resilience have used a variety of risk indicators, including prior achievement, experiences of homelessness, and dropping out of formal education (Rudd et al., 2021). The authors acknowledge the breadth of academic adversities that students may experience and overcome to demonstrate resilience. Future studies should look to expand the risk criteria to ensure that measures of academic resilience are firstly capturing those students who are experiencing acute or chronic adversity which heightens their risk of underachievement.

## Conclusion

Academic resilience has been operationalised in many ways within the definition-driven approach (Rudd et al., 2021; Tudor & Spray, 2018; Ye et al., 2021). This has complicated attempts to reconcile disparate findings about academic resilience. Accordingly, the current study sought to understand how these differences impact the resulting conclusions drawn about academic resilience. Two thresholds of risk and three thresholds of achievement commonly used in quantitative studies of resilience were employed to identify students demonstrating academic success despite adversity. Thus, the current study attempted to apply six operationalisations of academic resilience to three well-known ILSA datasets to examine the prevalence rates of and strength of protective factors related to academic resilience. Limitations related to missing data and the nature of the contextual data collected by each ILSA meant that the number of datasets used in the final set of analyses was much smaller than anticipated and required two different sets of protective factors to be explored depending on the ILSA being tested. The findings demonstrate that the decisions a researcher makes about how to operationalise academic resilience are important. Depending on the operationalisation employed, the prevalence rates of academic resilience varied from less than 5% to over 40% of all disadvantaged students. In contrast, the direction and strength of the protective factors studied remained much more consistent. The findings suggest that the use of relative risk and achievement thresholds, such as those currently used by PISA, provide the best indicator of the level of resilience within an at-risk group and can be used to explore how rates of resilience change over time.

By focussing on the Aotearoa NZ context, the current study investigated how academic resilience manifests in an education system that is currently producing inequitable outcomes for students. The large disparities between the country’s highest and lowest achievers, and the role that SES plays in perpetuating these disparities, were reflected in the consistently low rates of academic resilience produced by the relative thresholds of risk and achievement. Subject confidence was the most consistent predictor of academic resilience, while school SES, one of the two control variables explored in the current study, also predicted resilience. While the cross-sectional nature of ILSA data prevented the current study from establishing whether subject confidence facilitates resilience, or is a characteristic of resilient students, it is clear that this non-cognitive variable is important. Such findings demonstrate how the social, political, and economic dynamics of the local context have a strong bearing on how and how often academic resilience is demonstrated by the student population. In particular, if the prevalence rates of or strength of protective factors related to academic resilience are to be compared across contexts, the measure employed must acknowledge these local dynamics. Ultimately, any measure of academic resilience should capture students who are at a heightened risk of underachievement and who continue to achieve highly. To do this effectively, researchers must operationalise academic resilience in a way that is sensitive to and justified for the context being explored to ensure that students demonstrating academic resilience are accurately represented in contemporary research.

## Supplementary Information

Below is the link to the electronic supplementary material.

Supplementary file1 (PDF 632 KB)

## Data Availability

All data used in the current study, as well as the international large-scale assessment analysis tool, are publicly available on each of the relevant assessment centres’ websites (e.g. PISA: https://www.oecd.org/pisa/data/).

## Footnotes

1 OR = Odds ratio.

2 Aotearoa is the Māori name for New Zealand.

## Code availability

All data used in the current study, as well as the international large-scale assessment analysis tool, are publicly available on each of the relevant assessment centres’ websites (e.g. PISA: https://www.oecd.org/pisa/data/).
