# Supplementary material for: Investigating the measurement of academic resilience in Aotearoa New Zealand using international large-scale assessment data
Source: Educ Assess Eval Account. 2022 May 25;35(2):169–200. doi: 10.1007/s11092-022-09384-0 (PMC9131980; doi:10.1007/s11092-022-09384-0)
Supplement: Supplementary file 1 — Supplementary file1 (PDF 632 KB) [file 11092_2022_9384_MOESM1_ESM.pdf]

Appendix A

Table A1

Measures of educational resources used in the ILSA SES indices

Dataset	Number of items	Items
PISA	25	A desk to study at; a room of your own; a quiet place to study; a computer you can use for school work; educational software; a link to the Internet; classic literature; books of poetry; works of art; books to help with your school work; technical reference books; a dictionary; books on art, music, or design; a heat pump or air conditioning unit; boat; snowboard or skis; televisions; cars; bathrooms or ensuites; mobile phones with internet access; computers; tablet computers; Ebook readers; musical instruments; books
TIMSS		
Grade 4	3	Number of books in the home; number of children's books in the home; number of home study supports (internet connection; own room)
Grade 8	2	Number of books in the home; number of home study supports (internet connection; own room)
PIRLS	3	Number of books in the home; number of children's books in the home; number of home study supports (internet connection; own room)

Note. See technical documents outlined in Table 2 for more information about the SES indices.

Table A2

Operationalisation of the protective factors and control variables used in the current study

	PISA		TIMSS	
	Protective factor	Measurement	Protective factor	Measurement
Student-level	Subject confidence	2015: Science self-efficacy, higher scores reflect higher levels of self-efficacy, derived from 8 items; 2018: Reading competence, higher scores reflect higher levels of competence, derived from 3 items	Subject confidence	Mathematics: Higher scores reflect higher levels of confidence in mathematics, derived from 9 items; Science: Higher scores reflect higher levels of confidence in science, derived from 8 items
	Parental support	2015: Higher scores reflect higher levels of support, derived from 4 items; 2018: Higher scores reflect higher levels of support, derived from 3 items	Homework	Mathematics: Minutes spent completing mathematics homework, higher scores reflect more time taken, raw questionnaire item; Science: Minutes spent completing science homework, higher scores reflect more time taken, raw questionnaire item
	School belonging	Higher scores reflect higher levels of belonging,	School belonging	Higher scores reflect higher levels of belonging,

		derived from 6 items		derived from 5 items
School-level	Extracurricular activities	Higher scores reflect more extra-curricular activities offered, derived from 3 items	Curriculum implementation	Higher scores reflect lower levels of successful implementation; raw questionnaire item
	Student-teacher ratio	Higher scores reflect that each teacher is responsible for more students (i.e., larger class size), derived from 2 items	Teacher expectations	Higher scores reflect lower levels of teacher expectations; raw questionnaire item
	Professional development	Higher scores reflect a greater proportion of teaching staff attended a programme of professional development, raw questionnaire item	Parent expectations	Higher scores reflect lower levels of parent expectations; raw questionnaire item
Control variables	Gender	1: female, 2: male, raw questionnaire item	Gender	1: female, 2: male, raw questionnaire item
	School SES	Higher scores reflect that the school's roll of Year 10 students includes a higher proportion of students from 'socioeconomically disadvantaged homes', raw questionnaire item	School SES	Higher scores reflect that the school's roll includes a higher proportion of students from 'socioeconomically disadvantaged homes', raw questionnaire item

Appendix B

Table B1

Logistic regression models predicting academic resilience using relative risk threshold and absolute achievement thresholds (operationalisation number four)

Dataset	Predictors	Coefficient estimate	Standard error	Wald statistic	Odds ratio	Standard error	Significance
PISA 2015							
Reading (N = 7,637)	Intercept	-0.53	0.83	0.40	0.65	0.65	.53
	Control variables						
	Gender	0.64	0.21	8.91	1.90	0.42	.003**
	School SES	-0.02	0.01	10.81	0.98	0.01	.001**
	Student-level protective factors						
	Subject confidence	0.29	0.08	13.10	1.34	0.11	<.001***
	Parental support	0.12	0.10	1.58	1.13	0.11	.21
	School belonging	-0.02	0.13	0.01	0.99	0.13	.91
	School-level protective factors						
	Extracurricular activities	0.08	0.15	0.26	1.08	0.17	.61
	Student-teacher ratio	0.01	0.05	0.04	1.01	0.05	.84
	Professional development	0.01	0.00	2.34	1.01	0.00	.13
	Diagnostics						
	-2 Log Likelihood	976.16	766.66				
	Cox & Snell R Square	0.09	0.03				
Mathematics (N = 7,637)	Intercept	-0.48	0.83	0.34	0.71	0.70	.56
	Control variables						
	Gender	-0.10	0.24	0.18	0.91	0.22	.67
	School SES	-0.01	0.00	10.02	0.99	0.00	.002**
	Student-level protective factors						
	Subject confidence	0.19	0.07	6.52	1.21	0.09	.01*
	Parental support	0.00	0.10	.001	1.01	0.10	.97
	School belonging	-0.06	0.13	0.19	0.95	0.12	.66
	School-level protective factors						
	Extracurricular activities	0.02	0.19	.01	1.02	0.20	.93
	Student-teacher ratio	0.03	0.05	0.33	1.03	0.05	.57
	Professional development	0.00	0.00	0.37	1.00	0.00	.54
	Diagnostics						
	-2 Log Likelihood	9749.95	738.50				
	Cox & Snell R Square	0.05	0.02				

Science (N = 7,637)	Intercept	0.30	0.79	0.14	1.45	1.25	.71
	Control variables						
	Gender	-0.06	0.24	0.07	0.95	0.23	.80
	School SES	-0.02	0.00	11.22	0.98	0.00	.001**
	Student-level protective factors						
	Subject confidence	0.31	0.08	15.42	1.37	0.11	<.001***
	Parental support	0.06	0.09	0.48	1.07	0.10	.49
	School belonging	-0.09	0.12	0.57	0.91	0.11	.45
	School-level protective factors						
	Extracurricular activities	0.03	0.19	0.02	1.03	0.20	.89
	Student-teacher ratio	-0.01	0.04	0.13	0.99	0.04	.72
	Professional development	0.00	0.00	1.29	1.00	0.00	.26
	Diagnostics						
	-2 Log Likelihood	9918.53	755.09				
	Cox & Snell R Square	0.07	0.02				
PISA 2018							
Reading (N = 9,571)	Intercept	0.03	0.59	0.003	1.11	0.64	.96
	Control variables						
	Gender	0.36	0.20	3.45	1.44	0.29	.06
	School SES	-0.02	0.00	26.26	0.98	0.00	<.001***
	Student-level protective factors						
	Subject confidence	1.07	0.10	109.09	2.92	0.30	<.001***
	Parental support	0.00	0.08	0.00	1.00	0.08	.996
	School belonging	-0.12	0.10	1.28	0.89	0.09	.26
	School-level protective factors						
	Extracurricular activities	0.16	0.15	1.16	1.17	0.17	.28
	Student-teacher ratio	-0.01	0.03	0.09	0.99	0.03	.77
	Professional development	0.00	0.00	0.00	1.00	0.00	.99
	Diagnostics						
	-2 Log Likelihood	10977.15	479.68				
	Cox & Snell R Square	0.20	0.02				
Mathematics (N = 9,571)	Intercept	0.07	0.57	0.02	1.12	0.65	.90
	Control variables						
	Gender	-0.36	0.17	4.67	0.70	0.12	.03
	School SES	-0.02	0.00	18.80	0.98	0.00	<.001***
	Student-level protective factors						

	Subject confidence	0.60	0.09	39.46	1.82	0.17	<.001***
	Parental support	0.01	0.10	0.01	1.01	0.10	.93
	School belonging	-0.10	0.10	1.01	0.90	0.09	.32
	School-level protective factors						
	Extracurricular activities	0.11	0.14	0.53	1.11	0.16	.47
	Student-teacher ratio	-0.01	0.03	0.07	0.99	0.03	.79
	Professional development	0.00	0.00	0.14	1.00	0.00	.71
	Diagnostics						
	-2 Log Likelihood	11508.37	539.74				
	Cox & Snell R Square	0.12	0.03				
Science (N = 9,571)	Intercept	0.53	0.57	0.86	1.78	1.07	.36
	Control variables						
	Gender	-0.34	0.17	3.93	0.71	0.12	.05
	School SES	-0.02	0.00	33.89	0.98	0.00	<.001***
	Student-level protective factors						
	Subject confidence	0.86	0.10	74.88	2.36	0.24	<.001***
	Parental support	0.03	0.07	0.21	1.03	0.08	.64
	School belonging	-0.12	0.09	1.93	0.89	0.08	.16
	School-level protective factors						
	Extracurricular activities	0.09	0.19	0.23	1.10	0.21	.63
	Student-teacher ratio	-0.01	0.03	0.18	0.99	0.03	.67
	Professional development	0.00	0.00	0.04	1.00	0.00	.85
	Diagnostics						
	-2 Log Likelihood	11308.59	522.02				
	Cox & Snell R Square	0.17	0.03				
TIMSS 2015							
Mathematics (N = 10,289)	Intercept	-3.54	1.01	12.32	0.03	0.03	<.001***
	Control variables						
	Gender	0.19	0.18	1.15	1.21	0.22	.28
	School SES	-0.54	0.12	20.67	0.58	0.07	<.001***
	Student-level protective factors						
	Subject confidence	0.47	0.09	29.35	1.60	0.14	<.001***
	Homework	0.24	0.09	6.45	1.27	0.12	.01*
	School belonging	-0.01	0.06	0.04	0.99	0.06	.84
	School-level protective factors						

	Curriculum implementation	0.06	0.17	0.12	1.06	0.18	.73
	Teacher expectations	-0.39	0.21	3.51	0.68	0.14	.06
	Parent expectations	-0.24	0.14	2.66	0.79	0.11	.10
	Diagnostics						
	-2 Log Likelihood	10961.93	789.50				
	Cox & Snell R Square	0.18	0.04				
Science (N = 9,664)	Intercept	-0.77	0.77	1.00	0.47	0.39	.32
	Control variables						
	Gender	-0.07	0.17	0.18	0.93	0.16	.67
	School SES	-0.47	0.09	27.64	0.63	0.06	<.001***
	Student-level protective factors						
	Subject confidence	0.24	0.06	14.32	1.27	0.08	<.001***
	Homework	0.21	0.07	9.08	1.24	0.09	.003**
	School belonging	0.00	0.05	0.004	1.00	0.05	.95
	School-level protective factors						
	Curriculum implementation	-0.05	0.19	0.06	0.96	0.18	.80
	Teacher expectations	-0.38	0.20	3.42	0.69	0.14	.07
	Parent expectations	-0.18	0.13	1.92	0.84	0.11	.17
	Diagnostics						
	-2 Log Likelihood	11881.76	690.96				
	Cox & Snell R Square	0.12	0.03				
TIMSS 2019							
Mathematics (n = 9,748)	Intercept	-4.51	0.95	22.75	0.01	0.01	<.001***
	Control variables						
	Gender	0.33	0.23	2.10	1.39	0.32	.15
	School SES	-0.55	0.14	15.03	0.58	0.08	<.001***
	Student-level protective factors						
	Subject confidence	0.50	0.08	41.92	1.64	0.13	<.001***
	Homework	0.07	0.09	0.63	1.08	0.10	.43
	School belonging	0.06	0.06	0.83	1.06	0.07	.36
	School-level protective factors						
	Curriculum implementation	-0.23	0.21	1.20	0.79	0.17	.27
	Teacher expectations	-0.06	0.20	0.10	0.94	0.18	.76
	Parent expectations	0.01	0.19	0.001	1.01	0.19	.97
	Diagnostics						
	-2 Log Likelihood	10797.22	1087.58				

	Cox & Snell R Square	0.17	0.03				
Science (N = 9,366)	Intercept	-1.67	0.89	3.47	0.20	0.19	.06
	Control variables						
	Gender	0.04	0.23	0.03	1.04	0.24	.87
	School SES	-0.43	0.14	9.12	0.65	0.09	.003**
	Student-level protective factors						
	Subject confidence	0.22	0.08	7.95	1.25	0.10	.005**
	Homework	0.02	0.10	0.05	1.02	0.10	.82
	School belonging	0.04	0.06	0.54	1.04	0.06	.46
	School-level protective factors						
	Curriculum implementation	-0.03	0.24	0.01	0.98	0.24	.92
	Teacher expectations	-0.17	0.31	0.31	0.85	0.27	.58
	Parent expectations	0.10	0.20	0.26	1.11	0.22	.61
	Diagnostics						
	-2 Log Likelihood	11936.12	1026.02				
	Cox & Snell R Square	0.08	0.02				

*p < .05. **p < .01. ***p < .001.

Table B2

Logistic regression models predicting academic resilience using relative risk threshold and relative achievement thresholds (operationalisation number five)

Dataset	Predictors	Coefficient estimate	Standard error	Wald statistic	Odds ratio	Standard error	Significance
PISA 2015							
Reading (N = 7,637)	Intercept	-1.56	0.91	2.91	0.22	0.21	.09
	Control variables						
	Gender	0.69	0.26	7.05	2.01	0.55	.008**
	School SES	-0.02	0.01	11.02	0.98	0.01	.001**
	Student-level protective factors						
	Subject confidence	0.39	0.09	16.96	1.48	0.14	<.001***
	Parental support	0.04	0.12	0.13	1.05	0.13	.72
	School belonging	0.03	0.18	0.03	1.04	0.18	.87
	School-level protective factors						
	Extracurricular activities	-0.03	0.18	0.03	0.97	0.18	.87
	Student-teacher ratio	0.02	0.05	0.15	1.02	0.05	.70
	Professional development	0.00	0.01	0.90	1.00	0.01	.34
	Diagnostics						
	-2 Log Likelihood	7186.20	686.34				

	Cox & Snell R Square	0.08	0.03				
Mathematics (N = 7,637)	Intercept	-2.87	1.62	3.15	0.08	0.14	.08
	Control variables						
	Gender	-0.29	0.38	0.59	0.77	0.31	.44
	School SES	-0.02	0.01	4.86	0.98	0.01	.03*
	Student-level protective factors						
	Subject confidence	0.33	0.13	6.97	1.40	0.18	.008**
	Parental support	-0.07	0.17	0.17	0.94	0.16	.68
	School belonging	-0.02	0.21	0.01	0.99	0.21	.94
	School-level protective factors						
	Extracurricular activities	0.06	0.29	0.05	1.08	0.34	.83
	Student-teacher ratio	0.08	0.07	1.31	1.08	0.08	.25
	Professional development	0.00	0.01	0.01	1.00	0.01	.94
	Diagnostics						
	-2 Log Likelihood	4699.29	651.22				
	Cox & Snell R Square	0.04	0.02				
Science (N = 7,637)	Intercept	-2.91	1.20	5.93	0.06	0.09	.02*
	Control variables						
	Gender	-0.16	0.34	0.23	0.86	0.32	.63
	School SES	-0.02	0.01	6.73	0.98	0.01	.01*
	Student-level protective factors						
	Subject confidence	0.50	0.12	18.04	1.65	0.20	<.001***
	Parental support	0.02	0.13	0.03	1.02	0.14	.87
	School belonging	-0.13	0.22	0.33	0.89	0.20	.57
	School-level protective factors						
	Extracurricular activities	0.05	0.28	0.04	1.07	0.30	.84
	Student-teacher ratio	0.06	0.07	0.75	1.06	0.07	.39
	Professional development	0.01	0.01	0.77	1.01	0.01	.38
	Diagnostics						
	-2 Log Likelihood	4677.47	568.22				
	Cox & Snell R Square	0.05	0.02				
PISA 2018							
Reading (N = 9,571)	Intercept	-2.08	0.70	8.89	0.13	0.10	.003**
	Control variables						
	Gender	0.45	0.22	4.13	1.57	0.35	.04*
	School SES	-0.02	0.01	9.81	0.98	0.01	.002**

	Student-level protective factors						
	Subject confidence	0.94	0.12	56.53	2.56	0.33	<.001***
	Parental support	0.01	0.11	.004	1.01	0.11	.95
	School belonging	-0.15	0.11	2.02	0.86	0.09	.16
	School-level protective factors						
	Extracurricular activities	0.18	0.22	0.72	1.21	0.26	.40
	Student-teacher ratio	-0.01	0.04	0.07	0.99	0.04	.79
	Professional development	0.00	0.00	0.004	1.00	0.00	.95
	Diagnostics						
	-2 Log Likelihood	6293.06	477.14				
	Cox & Snell R Square	0.11	0.02				
Mathematics (N = 9,571)	Intercept	-1.92	0.82	5.52	0.16	0.14	.02*
	Control variables						
	Gender	-0.15	0.25	0.39	0.87	0.22	.53
	School SES	-0.02	0.01	10.09	0.98	0.01	.001**
	Student-level protective factors						
	Subject confidence	0.51	0.11	20.34	1.66	0.19	<.001***
	Parental support	0.04	0.12	0.14	1.05	0.13	.71
	School belonging	-0.07	0.12	0.39	0.93	0.11	.54
	School-level protective factors						
	Extracurricular activities	0.21	0.28	0.55	1.25	0.36	.46
	Student-teacher ratio	-0.01	0.04	0.04	0.99	0.04	.84
	Professional development	0.00	0.00	0.32	1.00	0.00	.57
	Diagnostics						
	-2 Log Likelihood	6440.26	468.04				
	Cox & Snell R Square	0.06	0.02				
Science (N = 9,571)	Intercept	-1.36	0.88	2.37	0.30	0.34	.12
	Control variables						
	Gender	-0.20	0.25	0.61	0.83	0.20	.44
	School SES	-0.02	0.01	13.86	0.98	0.01	<.001***
	Student-level protective factors						
	Subject confidence	0.81	0.12	44.93	2.24	0.27	<.001***
	Parental support	0.00	0.13	0.001	1.01	0.13	.97
	School belonging	-0.20	0.13	2.27	0.82	0.11	.13

	School-level protective factors						
	Extracurricular activities	0.22	0.26	0.71	1.25	0.32	.40
	Student-teacher ratio	-0.04	0.04	1.03	0.96	0.04	.31
	Professional development	0.00	0.00	.01	1.00	0.00	.93
	Diagnostics						
	-2 Log Likelihood	6248.11	523.97				
	Cox & Snell R Square	0.09	0.02				
TIMSS 2015 Mathematics (N = 10,289)	Intercept	-7.19	1.24	33.68	0.00	0.00	<.001***
	Control variables						
	Gender	0.17	0.29	0.32	1.19	0.34	.57
	School SES	-0.62	0.16	15.63	0.54	0.08	<.001***
	Student-level protective factors						
	Subject confidence	0.62	0.08	55.72	1.86	0.15	<.001***
	Homework	0.34	0.15	5.01	1.41	0.21	.03*
	School belonging	0.01	0.08	0.04	1.02	0.08	.85
	School-level protective factors						
	Curriculum implementation	-0.09	0.31	0.09	0.92	0.28	.77
	Teacher expectations	-0.63	0.36	2.98	0.54	0.20	.08
	Parent expectations	-0.13	0.25	0.26	0.89	0.23	.61
	Diagnostics						
	-2 Log Likelihood	4176.68	454.22				
	Cox & Snell R Square	0.12	0.03				
Science (N = 9,664)	Intercept	-4.83	1.56	9.59	0.01	0.02	.002**
	Control variables						
	Gender	0.12	0.41	0.08	1.16	0.45	.77
	School SES	-0.41	0.20	4.37	0.67	0.13	.04*
	Student-level protective factors						
	Subject confidence	0.35	0.09	15.38	1.42	0.13	<.001***
	Homework	0.27	0.14	3.81	1.31	0.18	.05
	School belonging	0.01	0.07	0.01	1.01	0.07	.91
	School-level protective factors						
	Curriculum implementation	-0.24	0.34	0.47	0.80	0.27	.49
	Teacher expectations	-0.46	0.41	1.29	0.65	0.26	.26
	Parent expectations	-0.03	0.30	0.01	0.99	0.30	.92
	Diagnostics						

	-2 Log Likelihood	3948.56	470.61				
	Cox & Snell R Square	0.05	0.01				
TIMSS 2019							
Mathematics (N = 9,748)	Intercept	-6.71	1.35	24.51	0.00	0.00	<.001***
	Control variables						
	Gender	0.08	0.38	0.04	1.10	0.43	.84
	School SES	-0.77	0.22	11.94	0.47	0.10	.001**
	Student-level protective factors						
	Subject confidence	0.64	0.09	51.00	1.90	0.17	<.001***
	Homework	0.12	0.09	1.74	1.13	0.11	.19
	School belonging	0.02	0.09	0.07	1.02	0.09	.79
	School-level protective factors						
	Curriculum implementation	-0.23	0.29	0.64	0.80	0.23	.42
	Teacher expectations	0.08	0.29	0.08	1.09	0.32	.78
	Parent expectations	-0.22	0.23	0.95	0.80	0.18	.33
	Diagnostics						
	-2 Log Likelihood	5584.46	806.52				
	Cox & Snell R Square	0.14	0.03				
Science (N = 9,366)	Intercept	-4.18	1.41	8.75	0.02	0.03	.003**
	Control variables						
	Gender	-0.34	0.33	1.06	0.72	0.23	.30
	School SES	-0.51	0.17	8.82	0.60	0.10	.003**
	Student-level protective factors						
	Subject confidence	0.35	0.10	13.59	1.42	0.14	<.001***
	Homework	0.06	0.14	0.17	1.06	0.15	.69
	School belonging	0.05	0.09	0.40	1.06	0.09	.53
	School-level protective factors						
	Curriculum implementation	-0.48	0.30	2.58	0.63	0.19	.11
	Teacher expectations	-0.02	0.32	.003	0.99	0.33	.95
	Parent expectations	0.00	0.24	0.00	1.00	0.24	.10
	Diagnostics						
	-2 Log Likelihood	5094.39	680.56				
	Cox & Snell R Square	0.07	0.02				

**p* < .05. ***p* < .01. ****p* < .001.

Table B3

Logistic regression models predicting academic resilience using relative risk threshold and resilience residuals achievement thresholds (operationalisation number six)

Dataset	Predictors	Coefficient estimate	Standard error	Wald statistic	Odds ratio	Standard error	Significance
PISA 2015							
Reading (N = 7,637)	Intercept	-1.04	0.82	1.59	0.35	0.34	.21
	Control variables						
	Gender	0.74	0.18	16.18	2.09	0.40	<.001***
	School SES	-0.02	0.01	15.99	0.98	0.01	<.001***
	Student-level protective factors						
	Subject confidence	0.28	0.09	10.82	1.33	0.12	.001**
	Parental support	0.01	0.08	0.01	1.01	0.08	.92
	School belonging	0.13	0.13	1.04	1.14	0.14	.31
	School-level protective factors						
	Extracurricular activities	0.11	0.16	0.46	1.11	0.18	.50
	Student-teacher ratio	-0.01	0.05	0.09	0.99	0.05	.77
	Professional development	0.00	0.00	1.23	1.00	0.00	.27
	Diagnostics						
	-2 Log Likelihood	7854.89	678.09				
	Cox & Snell R Square	0.09	0.03				
Mathematics (N = 7,637)	Intercept	-1.32	0.76	3.01	0.27	0.20	.08
	Control variables						
	Gender	-0.08	0.18	0.21	0.92	0.17	.65
	School SES	-0.02	0.00	19.93	0.98	0.00	<.001***
	Student-level protective factors						
	Subject confidence	0.32	0.09	13.28	1.37	0.12	<.001***
	Parental support	-0.08	0.09	0.84	0.92	0.08	.36
	School belonging	-0.16	0.14	1.41	0.85	0.11	.24
	School-level protective factors						
	Extracurricular activities	-0.19	0.18	1.11	0.83	0.16	.29
	Student-teacher ratio	0.09	0.04	4.52	1.09	0.05	.03*
	Professional development	0.00	0.00	0.09	1.00	0.00	.76
	Diagnostics						
	-2 Log Likelihood	7857.12	643.58				
	Cox & Snell R Square	0.07	0.02				

Science (N = 7,637)	Intercept	-1.00	0.84	1.43	0.37	0.31	.23
	Control variables						
	Gender	-0.16	0.21	0.57	0.86	0.18	.45
	School SES	-0.02	0.01	11.11	0.98	0.01	.001**
	Student-level protective factors						
	Subject confidence	0.37	0.07	24.95	1.45	0.11	<.001***
	Parental support	-0.10	0.10	0.97	0.91	0.09	.33
	School belonging	-0.23	0.14	2.87	0.79	0.11	.09
	School-level protective factors						
	Extracurricular activities	-0.11	0.18	0.40	0.89	0.16	.53
	Student-teacher ratio	0.06	0.05	1.37	1.06	0.05	.24
	Professional development	0.00	0.00	0.01	1.00	0.00	.94
	Diagnostics						
	-2 Log Likelihood	7954.08	678.77				
	Cox & Snell R Square	0.07	0.02				
PISA 2018							
Reading (N = 9,571)	Intercept	-1.18	0.52	5.06	0.31	0.16	.03*
	Control variables						
	Gender	0.56	0.17	11.36	1.75	0.30	.001**
	School SES	-0.02	0.00	17.70	0.98	0.00	<.001***
	Student-level protective factors						
	Subject confidence	1.00	0.09	132.76	2.71	0.23	<.001***
	Parental support	-0.08	0.08	1.01	0.92	0.07	.31
	School belonging	-0.09	0.09	0.96	0.91	0.08	.33
	School-level protective factors						
	Extracurricular activities	0.16	0.12	1.81	1.18	0.14	.18
	Student-teacher ratio	0.00	0.03	0.02	1.00	0.03	.89
	Professional development	0.00	0.00	0.04	1.00	0.00	.85
	Diagnostics						
	-2 Log Likelihood	9486.69	427.79				
	Cox & Snell R Square	0.17	0.02				
Mathematics (N = 9,571)	Intercept	-1.09	0.48	5.21	0.34	0.15	.02*
	Control variables						
	Gender	-0.13	0.14	0.85	0.88	0.13	.36
	School SES	-0.02	0.00	28.52	0.98	0.00	<.001***
	Student-level protective factors						

	Subject confidence	0.54	0.08	44.62	1.71	0.14	<.001***
	Parental support	-0.03	0.08	0.14	0.97	0.08	.71
	School belonging	-0.02	0.08	0.06	0.98	0.08	.80
	School-level protective factors						
	Extracurricular activities	0.06	0.13	0.18	1.06	0.14	.67
	Student-teacher ratio	0.02	0.03	0.83	1.02	0.03	.36
	Professional development	0.00	0.00	2.03	1.00	0.00	.15
	Diagnostics						
	-2 Log Likelihood	10054.57	463.66				
	Cox & Snell R Square	0.09	0.02				
Science (N = 9,571)	Intercept	0.06	0.41	0.02	1.06	0.46	.88
	Control variables						
	Gender	-0.32	0.14	5.01	0.73	0.11	.03*
	School SES	-0.02	0.00	32.56	0.98	0.00	<.001***
	Student-level protective factors						
	Subject confidence	0.72	0.09	67.45	2.06	0.18	<.001***
	Parental support	0.10	0.08	1.69	1.11	0.09	.19
	School belonging	-0.20	0.08	6.13	0.82	0.07	.01**
	School-level protective factors						
	Extracurricular activities	0.05	0.13	0.17	1.06	0.14	.68
	Student-teacher ratio	-0.02	0.02	1.14	0.98	0.02	.29
	Professional development	0.00	0.00	0.01	1.00	0.00	.91
	Diagnostics						
	-2 Log Likelihood	9963.91	436.49				
	Cox & Snell R Square	0.13	0.02				
TIMSS 2015							
Mathematics (N = 10,289)	Intercept	-5.24	1.04	25.19	0.01	0.01	<.001***
	Control variables						
	Gender	0.15	0.15	1.09	1.17	0.17	.30
	School SES	-0.49	0.10	21.95	0.61	0.06	<.001***
	Student-level protective factors						
	Subject confidence	0.52	0.07	52.20	1.69	0.12	<.001***
	Homework	0.15	0.08	3.11	1.16	0.10	.08
	School belonging	0.04	0.04	1.33	1.04	0.04	.25
	School-level protective factors						

	Curriculum implementation	-0.18	0.17	1.13	0.83	0.14	.29
	Teacher expectations	-0.21	0.19	1.22	0.81	0.16	.27
	Parent expectations	-0.05	0.15	0.10	0.95	0.14	.75
	Diagnostics						
	-2 Log Likelihood	9670.82	672.38				
	Cox & Snell R Square	0.15	0.02				
Science (N = 9,664)	Intercept	-1.75	0.60	8.49	0.17	0.10	.004**
	Control variables						
	Gender	-0.05	0.12	0.14	0.96	0.12	.71
	School SES	-0.29	0.10	8.23	0.75	0.08	.001**
	Student-level protective factors						
	Subject confidence	0.22	0.06	12.83	1.24	0.08	<.001***
	Homework	0.19	0.07	6.80	1.21	0.09	.009**
	School belonging	-0.02	0.04	0.33	0.98	0.04	.57
	School-level protective factors						
	Curriculum implementation	-0.14	0.16	0.75	0.87	0.14	.39
	Teacher expectations	-0.34	0.19	3.23	0.72	0.14	.07
	Parent expectations	0.07	0.10	0.43	0.94	0.09	.51
	Diagnostics						
	-2 Log Likelihood	10185.08	567.29				
	Cox & Snell R Square	0.06	0.01				
TIMSS 2019							
Mathematics (N = 9,748)	Intercept	-2.10	0.69	9.29	0.12	0.08	.002**
	Control variables						
	Gender	0.14	0.18	0.58	1.15	0.21	.45
	School SES	-0.26	0.11	6.13	0.77	0.08	.01*
	Student-level protective factors						
	Subject confidence	0.22	0.06	14.10	1.24	0.07	<.001***
	Homework	0.07	0.07	1.04	1.07	0.07	.31
	School belonging	0.00	0.04	0.00	1.00	0.04	.99
	School-level protective factors						
	Curriculum implementation	-0.11	0.17	0.41	0.90	0.15	.52
	Teacher expectations	-0.07	0.15	0.21	0.93	0.14	.65
	Parent expectations	-0.04	0.19	0.04	0.96	0.18	.85
	Diagnostics						
	-2 Log Likelihood	11579.33	1084.73				

	Cox & Snell R Square	0.05	0.02				
Science (N = 9,366)	Intercept	-2.30	0.88	6.89	0.10	0.09	.009**
	Control variables						
	Gender	0.11	0.18	0.35	1.11	0.20	.56
	School SES	-0.26	0.11	5.93	0.77	0.08	.02*
	Student-level protective factors						
	Subject confidence	0.23	0.07	9.72	1.26	0.09	.002**
	Homework	0.00	0.07	0.00	1.00	0.07	.97
	School belonging	0.01	0.05	0.04	1.01	0.05	.84
	School-level protective factors						
	Curriculum implementation	-0.04	0.16	0.05	0.96	0.15	.82
	Teacher expectations	-0.04	0.15	0.07	0.96	0.15	.80
	Parent expectations	-0.02	0.18	0.01	0.98	0.18	.93
	Diagnostics						
	-2 Log Likelihood	11207.32	1004.89				
	Cox & Snell R Square	0.04	0.02				

*p < .05. **p < .01. ***p < .001.

Table B4

Logistic regression models predicting academic resilience using absolute risk threshold and absolute achievement thresholds (operationalisation number one)

Dataset	Predictors	Coefficient estimate	Standard error	Wald statistic	Odds ratio	Standard error	Significance
TIMSS 2015							
Mathematics (N = 2,489)	Intercept	-7.26	3.03	5.73	0.00	0.01	.02*
	Control variables						
	Gender	-0.08	0.52	0.03	0.96	0.52	.87
	School SES	-0.33	0.23	1.98	0.72	0.17	.16
	Student-level protective factors						
	Subject confidence	0.83	0.26	10.55	2.32	0.63	.001**
	Homework	0.11	0.19	0.34	1.12	0.21	.56
	School belonging	-0.03	0.12	0.07	0.97	0.11	.80
	School-level protective factors						
	Curriculum implementation	0.01	0.43	0.00	1.03	0.45	.98
	Teacher expectations	-0.55	0.53	1.09	0.59	0.33	.30
	Parent expectations	-0.20	0.37	0.28	0.84	0.31	.60
	Diagnostics						

	-2 Log Likelihood	2106.21	298.17				
	Cox & Snell R Square	0.20	0.06				
Science (N = 2,331)	Intercept	-3.33	1.47	5.15	0.05	0.07	.02*
	Control variables						
	Gender	-0.22	0.35	0.41	0.80	0.29	.52
	School SES	-0.24	0.22	1.24	0.79	0.17	.27
	Student-level protective factors						
	Subject confidence	0.38	0.15	6.03	1.46	0.23	.01*
	Homework	0.31	0.22	1.99	1.38	0.30	.16
	School belonging	-0.03	0.12	0.07	0.97	0.11	.80
	School-level protective factors						
	Curriculum implementation	0.11	0.35	0.10	1.13	0.40	.76
	Teacher expectations	-0.38	0.45	0.71	0.70	0.32	.40
	Parent expectations	-0.16	0.27	0.34	0.86	0.24	.56
	Diagnostics						
	-2 Log Likelihood	2617.00	285.86				
	Cox & Snell R Square	0.10	0.05				
TIMSS 2019							
Mathematics (N = 2,172)	Intercept	-2.01	2.25	0.80	0.18	0.52	.37
	Control variables						
	Gender	0.33	0.44	0.56	1.41	0.64	.45
	School SES	-0.70	0.25	7.65	0.50	0.13	.006**
	Student-level protective factors						
	Subject confidence	0.46	0.20	5.27	1.59	0.34	.02*
	Homework	-0.17	0.26	0.42	0.85	0.22	.52
	School belonging	-0.06	0.15	0.15	0.94	0.15	.70
	School-level protective factors						
	Curriculum implementation	-0.20	0.44	0.21	0.83	0.37	.65
	Teacher expectations	-0.05	0.46	0.01	0.98	0.47	.91
	Parent expectations	-0.18	0.51	0.13	0.87	0.46	.72
	Diagnostics						
	-2 Log Likelihood	2303.91	402.19				

	Cox & Snell R Square	0.15	0.06				
Science (N = 2,100)	Intercept	0.45	2.29	0.04	2.31	7.15	.84
	Control variables						
	Gender	0.03	0.48	0.01	1.06	0.56	.95
	School SES	-0.34	0.20	2.90	0.71	0.14	.09
	Student-level protective factors						
	Subject confidence	0.26	0.18	2.21	1.30	0.23	.14
	Homework	-0.09	0.29	0.10	0.93	0.27	.76
	School belonging	-0.12	0.15	0.60	0.89	0.13	.44
	School-level protective factors						
	Curriculum implementation	0.05	0.44	0.12	1.08	0.47	.91
	Teacher expectations	-0.25	0.52	0.23	0.81	0.44	.63
	Parent expectations	-0.50	0.36	1.90	0.61	0.23	.17
	Diagnostics						
	-2 Log Likelihood	2438.60	373.89				
	Cox & Snell R Square	0.12	0.07				

*$p < .05$. **$p < .01$. ***$p < .001$.

Table B5

Logistic regression models predicting academic resilience using absolute risk threshold and relative achievement thresholds (operationalisation number two)

Dataset	Predictors	Coefficient estimate	Standard error	Wald statistic	Odds ratio	Standard error	Significance
TIMSS 2015 Mathematics (N = 2,489)	Intercept	-9.11	3.76	5.87	0.00	0.00	.02*
	Control variables						
	Gender	-0.24	0.77	0.10	0.79	0.65	.75
	School SES	-1.23	0.82	2.26	0.31	0.23	.13
	Student-level protective factors						
	Subject confidence	0.60	0.25	5.86	1.83	0.47	.02*
	Homework	0.63	0.51	1.54	1.89	0.94	.21
	School belonging	-0.01	0.22	0.00	1.00	0.23	.98
	School-level protective factors						
	Curriculum implementation	1.04	1.11	0.87	3.13	4.58	.35
	Teacher expectations	-0.96	1.17	0.66	0.41	0.58	.42

	Parent expectations	-0.01	0.72	0.00	0.99	0.78	.99
	Diagnostics						
	-2 Log Likelihood	539.37	165.66				
	Cox & Snell R Square	0.09	0.05				
Science (N = 2,331)	Intercept	-9.76	3.91	6.23	0.00	0.00	.01*
	Control variables						
	Gender	0.27	1.07	0.06	1.46	1.60	.80
	School SES	-0.17	0.54	0.10	0.87	0.47	.75
	Student-level protective factors						
	Subject confidence	0.30	0.31	0.98	1.37	0.41	.32
	Homework	1.19	0.36	11.11	3.30	1.20	.001**
	School belonging	0.04	0.22	0.03	1.05	0.23	.86
	School-level protective factors						
	Curriculum implementation	-0.46	0.91	0.26	0.65	0.65	.61
	Teacher expectations	-0.46	1.29	0.13	0.81	1.34	.72
	Parent expectations	0.41	0.63	0.43	1.58	0.41	.32
	Diagnostics						
	-2 Log Likelihood	488.13	168.22				
	Cox & Snell R Square	0.06	0.04				
TIMSS 2019							
Mathematics (N = 2,172)	Intercept	-4.69	4.05	1.34	0.02	0.08	.25
	Control variables						
	Gender	-0.24	0.92	0.07	0.89	0.86	.79
	School SES	-0.69	0.54	1.62	0.50	0.28	.20
	Student-level protective factors						
	Subject confidence	0.84	0.37	5.22	2.33	1.01	.02*
	Homework	0.19	0.40	0.23	1.24	0.51	.63
	School belonging	-0.29	0.24	1.42	0.75	0.18	.23
	School-level protective factors						
	Curriculum implementation	-0.78	0.84	0.85	0.48	0.42	.36
	Teacher expectations	-0.48	0.69	0.50	0.63	0.48	.48
	Parent expectations	-0.14	0.80	0.03	0.92	0.84	.86
	Diagnostics						

	-2 Log Likelihood	980.60	309.50				
	Cox & Snell R Square	0.13	0.06				
Science (N = 2,100)	Intercept	0.12	4.11	0.00	6.16	32.27	.98
	Control variables						
	Gender	0.12	0.93	0.02	1.22	1.13	.90
	School SES	-0.40	0.41	0.95	0.68	0.29	.33
	Student-level protective factors						
	Subject confidence	0.35	0.27	1.66	1.43	0.40	.20
	Homework	-0.69	0.50	1.91	0.52	0.27	.17
	School belonging	-0.15	0.27	0.33	0.87	0.25	.56
	School-level protective factors						
	Curriculum implementation	-0.71	0.84	0.72	0.54	0.44	.40
	Teacher expectations	0.30	0.95	0.10	1.51	1.33	.75
	Parent expectations	-1.02	0.66	2.40	0.39	0.28	.12
	Diagnostics						
	-2 Log Likelihood	829.39	295.56				
	Cox & Snell R Square	0.09	0.04				

*$p < .05$. **$p < .01$. ***$p < .001$.

Table B6

Logistic regression models predicting academic resilience using absolute risk threshold and resilience residuals achievement thresholds (operationalisation number three)

Dataset	Predictors	Coefficient estimate	Standard error	Wald statistic	Odds ratio	Standard error	Significance
TIMSS 2015 Mathematics (N = 2,489)	Intercept	-5.77	2.13	7.32	0.00	0.01	.007**
	Control variables						
	Gender	0.03	0.36	0.01	1.03	0.37	.93
	School SES	-0.25	0.18	1.79	0.78	0.14	.18
	Student-level protective factors						
	Subject confidence	0.53	0.14	14.33	1.70	0.24	<.001***
	Homework	-0.05	0.19	0.07	0.95	0.18	.80
	School belonging	0.13	0.08	2.62	1.14	0.09	.11
	School-level protective factors						
	Curriculum implementation	0.03	0.27	0.01	1.03	0.29	.93

	Teacher expectations	-0.21	0.39	0.29	0.81	0.32	.59
	Parent expectations	-0.19	0.27	0.49	0.82	0.23	.48
	Diagnostics						
	-2 Log Likelihood	2741.89	351.02				
	Cox & Snell R Square	0.13	0.05				
Science (N = 2,331)	Intercept	-3.44	1.27	7.31	0.03	0.04	.007**
	Control variables						
	Gender	-0.21	0.33	0.40	0.81	0.27	.53
	School SES	-0.02	0.16	0.01	0.99	0.16	.93
	Student-level protective factors						
	Subject confidence	0.34	0.14	6.10	1.40	0.19	.01*
	Homework	0.42	0.19	4.76	1.53	0.30	.03*
	School belonging	-0.06	0.09	0.51	0.94	0.08	.48
	School-level protective factors						
	Curriculum implementation	0.05	0.22	0.05	1.05	0.23	.83
	Teacher expectations	-0.43	0.32	1.78	0.65	0.22	.18
	Parent expectations	-0.06	0.18	0.13	0.94	0.17	.72
	Diagnostics						
	-2 Log Likelihood	2676.43	246.33				
	Cox & Snell R Square	0.09	0.05				
TIMSS 2019							
Mathematics (N = 2,172)	Intercept	0.72	1.70	0.18	2.06	3.73	.67
	Control variables						
	Gender	-0.09	0.33	0.07	0.92	0.31	.80
	School SES	-0.33	0.17	3.57	0.72	0.13	.06
	Student-level protective factors						
	Subject confidence	0.18	0.14	1.56	1.20	0.17	.21
	Homework	0.02	0.14	0.01	1.02	0.15	.91
	School belonging	-0.17	0.11	2.23	0.84	0.10	.14
	School-level protective factors						
	Curriculum implementation	0.12	0.29	0.18	1.13	0.33	.67
	Teacher expectations	-0.13	0.29	0.20	0.88	0.26	.66

	Parent expectations	-0.27	0.29	0.84	0.76	0.22	.36
	Diagnostics						
	-2 Log Likelihood	2713.87	398.90				
	Cox & Snell R Square	0.07	0.04				
Science (N = 2,100)	Intercept	0.54	1.90	0.08	1.72	3.60	.78
	Control variables						
	Gender	-0.27	0.42	0.43	0.76	0.32	.51
	School SES	-0.21	0.19	1.32	0.81	0.15	.25
	Student-level protective factors						
	Subject confidence	0.12	0.15	0.64	1.12	0.16	.43
	Homework	-0.02	0.17	0.01	0.98	0.17	.93
	School belonging	-0.07	0.11	0.40	0.93	0.10	.53
	School-level protective factors						
	Curriculum implementation	0.10	0.25	0.16	1.11	0.28	.69
	Teacher expectations	-0.21	0.24	0.77	0.81	0.20	.38
	Parent expectations	-0.26	0.29	0.75	0.77	0.23	.39
	Diagnostics						
	-2 Log Likelihood	2689.53	380.47				
	Cox & Snell R Square	0.04	0.04				

*$p < .05$. **$p < .01$. ***$p < .001$.
